# Gut microbiota profiles associated with temporal lobe epilepsy and psychiatric comorbidities: a family-matched case–control 16S rRNA study

**DOI:** 10.1186/s12883-026-04958-7

**Published:** 2026-05-12

**Authors:** Chengyan Song, Yajun Li, Yanchun Deng, Donghong He, Xinhui Fan

**Affiliations:** 1 Department of Neurology, Yulin Hospital, First Affiliated Hospital of Xi’an Jiaotong University, Yulin, Shaanxi 719000 China; 2 Department of Neurology, Northwest Women’s and Children’s Hospital, Xi’an, Shaanxi 710003 China; 3 Department of Neurology, Xijing Hospital, Xi’an, Shaanxi 710032 China

**Keywords:** Gut microbiota, Temporal lobe epilepsy, Psychiatric comorbidity, ANCOM-BC, 16S rRNA, Drug-resistant epilepsy

## Abstract

We investigated alterations in the intestinal microbiota of patients with temporal lobe epilepsy (TLE) and their associations with drug resistance and psychiatric comorbidities. Thirty TLE patients and 30 family-matched healthy controls sharing the same household diet were recruited, and fecal samples were analyzed by high-throughput 16S rDNA sequencing on the Illumina MiSeq $2\times 300$ bp platform. Differential abundance was assessed using Metastats and LEfSe with Benjamini–Hochberg false-discovery-rate correction, and independently validated using ANCOM-BC to account for the compositional nature of microbiome data. Community $\alpha$- and $\beta$-diversity indices showed no significant differences between groups; however, ANCOM-BC identified species-level signatures in drug-resistant epilepsy, including significant depletion of *Bacteroides plebeius* and *Coprococcus comes*. Among psychiatric subgroups, *Ruminococcus* was significantly reduced in patients with comorbid depression, while *Bilophila* was enriched in those with comorbid anxiety and depression. *Bacteroides stercoris* distinguished the anxiety-plus-depression subgroup from the depression-only subgroup with robust support from both ANCOM and ANCOM-BC. Given the modest overall sample size ($n=30$ per arm) and small psychiatric and drug-resistance subgroups, these findings should be regarded as exploratory and hypothesis-generating associations rather than definitive biomarkers. They identify candidate microbial taxa warranting validation in larger, longitudinal cohorts combined with metagenomic and metabolomic approaches.

**Supplementary Information:**

The online version contains supplementary material available at 10.1186/s12883-026-04958-7.

## Introduction

The intestinal microbiota has been dubbed the “second brain” because of its extensive involvement in human physiology and pathology. Epilepsy, a common chronic brain disorder, profoundly affects physical and mental health and imposes a heavy disease burden. Comorbid anxiety and depression complicate diagnosis and therapy. Previous studies have linked gut dysbiosis to the etiology of epilepsy and affective disorders and suggest that restoring normal microbiota improves patient outcomes [1]. Similar mechanisms—including neurotransmitter imbalances, immune dysregulation and genetic factors—may underpin epilepsy and mood disorders. Based on these shared mechanistic pathways, we hypothesized that patients with TLE and comorbid anxiety/depression would exhibit distinct gut microbiota profiles associated with their clinical phenotype. However, interactions between epilepsy-associated psychiatric disorders and gut microbiota have not been adequately studied. Therefore we used high-throughput sequencing and bioinformatics to analyze fecal samples from patients with epilepsy, epilepsy with psychiatric comorbidity and healthy controls, aiming to delineate microbiota differences, explore relationships with seizure severity and psychiatric status and provide evidence for novel therapeutic strategies.

## Materials and methods

### Reagents and instruments

 

- Phusion High-Fidelity PCR Master Mix (New England Biolabs, Hitchin, UK)
- UltraSYBR Mixture (ComWin Biotech, Beijing, China)
- FTC-3000™ Real-Time PCR System (Fengling, Shanghai, China)
- Precision Electronic Balance (Zhuojing, Shanghai, China)
- MiSeq Reagent Kits v3 (Illumina, San Diego, CA, USA)
- Vortex-5 Mixer (QLiNBeIER, Haimen, China)
- Electronic Constant-Temperature Stainless Steel Water Bath HHS-2S (Shanghai, China)
- Eppendorf Centrifuge (Eppendorf, Hamburg, Germany)
- Gel Electrophoresis and Imaging System (Bio-Rad, Hercules, CA, USA)
- ABI 9700 Thermal Cycler (Applied Biosystems, Foster City, CA, USA)
- Axygen Gel Extraction Kit (Axygen, Union City, CA, USA)
- MiSeq Sequencer (Illumina, San Diego, CA, USA)
- DNA Markers DL9000 (Xinbainuo, Beijing, China) and DL2000 (Takara, Shiga, Japan)

### Clinical data and grouping

Thirty patients with temporal-lobe epilepsy diagnosed in the neurology department of Xijing Hospital from November 2023 to January 2024 were recruited. Diagnosis and classification conformed to the 2017 International League Against Epilepsy (ILAE) classification of the epilepsies [2], and psychiatric diagnoses followed the CCMD-3. Based on seizure control status, patients were divided into drug-resistant epilepsy (RE; $n=7$) and non-drug-resistant epilepsy (NRE; $n=23$). According to National Hospital Seizure Severity Scale (NHS3) [3] scores they were grouped into NHS3-1 (8 patients), NHS3-2 (13 patients) and NHS3-3 (9 patients). Anxiety and depression were assessed with the Hamilton Anxiety Rating Scale (HAMA) [4], Hamilton Depression Rating Scale (HAMD) [5], Self-rating Anxiety Scale (SAS) [6] and Self-rating Depression Scale (SDS) [7], applying the following predefined thresholds derived from each scale’s original validation study: anxiety was defined as HAMA $\ge 14$ or SAS standardized score $\ge 50$; depression was defined as HAMD $\ge 8$ or SDS standardized score $\ge 53$; the comorbid anxiety–depression group required fulfillment of both anxiety and depression criteria. Patients were assigned to B1 (without anxiety/depression; 7 patients), B2 (depression; 5 patients) and B3 (anxiety + depression; 18 patients). Thirty healthy family members sharing the same household served as controls (CON). Because controls were recruited from the same household as TLE participants, they shared the same habitual diet, which partially controls for dietary confounders; however, formal dietary assessment (e.g., food-frequency questionnaire) was not performed, and this is acknowledged as a limitation.

### Inclusion and exclusion criteria

Inclusion criteria (TLE group): (i) age 18–65 years; (ii) clinical, electroencephalographic, and neuroimaging diagnosis of temporal-lobe epilepsy per ILAE 2017 criteria; (iii) a stable antiseizure medication (ASM) regimen for at least 3 months prior to enrollment; (iv) ability to provide written informed consent. Inclusion criteria (control group): healthy family members (spouse or first-degree relative) sharing the same household and habitual diet as a TLE participant, age-matched within $\pm 5$ years. Exclusion criteria applied to both groups: (i) use of systemic antibiotics or probiotics/prebiotics within 4 weeks before fecal sampling; (ii) history of inflammatory bowel disease, irritable bowel syndrome, or other significant gastrointestinal disease; (iii) diabetes mellitus, malignancy, autoimmune disease, or other major chronic medical illness known to alter gut microbiota; (iv) pregnancy or lactation; (v) major dietary change (e.g., initiation of a vegetarian or ketogenic diet) within the preceding 3 months; (vi) regular alcohol consumption ($>14$ units/week).

### Sample size and statistical power

This was an exploratory case–control study. An *a priori* sample-size estimate was derived from previously published epilepsy microbiome studies [8], which reported moderate effect sizes (Cohen’s $d \approx 0.7$–1.0) for key discriminative genera between epilepsy and control groups. With $n=30$ per group, the primary TLE-vs-CON comparison has approximately 80% power to detect a two-group difference with $d \ge 0.75$ at $\alpha = 0.05$ (two-sided). We explicitly acknowledge that subgroup analyses—in particular the drug-resistant ($n=7$) and B2 depression-only ($n=5$) strata—are underpowered for formal hypothesis testing and are reported as exploratory, hypothesis-generating findings rather than as definitive results.

### Fecal sample collection

Fresh mid-portion stool ($\ge 2$ g) was collected using sterile collection kits. Samples were placed in 3 mL sterile tubes, avoiding contamination, and were transferred to a $-80^{\circ }\textrm{C}$ freezer within 24 h.

### DNA extraction from fecal samples

Microbial DNA from fecal samples was extracted using the QIAamp DNA Stool Mini Kit (QIAGEN, Hilden, Germany).

1. The frozen fecal samples were thawed, and 0.18–0.22 g of each sample was weighed using a precision electronic balance, placed into centrifuge tubes, and handled on ice.
2. According to the manufacturer’s protocol of the DNA extraction kit, genomic DNA was extracted from all samples using the provided reagents and instruments in a stepwise and rapid manner.
3. The integrity of the extracted genomic DNA was assessed by 1.2% agarose gel electrophoresis.
4. All intact genomic DNA samples were stored at $-20\,^{\circ }\textrm{C}$ for subsequent sequencing and analysis.

### Bacterial 16S rDNA PCR amplification and MiSeq sequencing

The V4–V5 region of the bacterial 16S rDNA was amplified using the universal primers 515 F (5’-GTGCCAGCMGCCGCGG-3’) and 926R (5’-CCGTCAATTCMTTTGAGTTT-3’). The PCR products were examined by 1.2% agarose gel electrophoresis. Samples with satisfactory amplification were excised from a 2% agarose gel, and the recovered products were used as templates for an additional 8-cycle PCR to add the sequencing adapters, primers, and index sequences required for the Illumina platform. Sequencing was then performed on the Illumina MiSeq platform (2 $\times$ 300 bp).

The PCR amplification was conducted in two steps:

1. *First PCR*: The reaction mixture (50 $\mu$L total volume) contained 10 $\mu$L of 5$\times$ buffer, 1 $\mu$L of dNTPs (10 mM), 1 U of Phusion High-Fidelity DNA polymerase, 1 $\mu$L each of forward and reverse primers (10 mM), 20–50 ng of template DNA, and nuclease-free water to volume. The PCR cycling conditions were: initial denaturation at 94 $^{\circ }$C for 2 min; followed by 25 cycles of 94 $^{\circ }$C for 30 s, 56 $^{\circ }$C for 30 s, 72 $^{\circ }$C for 30 s; and a final extension at 72 $^{\circ }$C for 5 min.
2. *Second PCR*: The reaction mixture (40 $\mu$L total volume) contained 8 $\mu$L of 5$\times$ buffer, 1 $\mu$L each of dNTPs (10 mM), 5 $\mu$L of template DNA, and nuclease-free water to volume. The PCR cycling conditions were: 94 $^{\circ }$C for 30 s, 56 $^{\circ }$C for 30 s, 72 $^{\circ }$C for 30 s, followed by a final extension at 72 $^{\circ }$C for 5 min and hold at 10 $^{\circ }$C, for a total of 8 cycles.

The amplified bacterial 16S rDNA V4–V5 region was subsequently sequenced according to the experimental requirements.

### Sequencing data processing

Barcode sequences were added to the raw sequencing reads to obtain valid sequences. Low-quality bases at the ends of the valid sequences were trimmed using Trimmomatic software, and paired-end reads were merged into complete sequences using FLASH software. Finally, sequence quality control and filtering were performed with the Mothur software package to remove low-quality bases, sequences that did not meet length requirements, and chimeric sequences generated during PCR amplification, thereby obtaining optimized sequences.

### OTU clustering and taxonomic annotation

All optimized sequences were clustered into operational taxonomic units (OTUs) at a 97% similarity threshold using the UPARSE software. Representative sequences of each OTU were aligned against the SILVA123 database for taxonomic annotation. Based on the taxonomic classification, the microbial community structure was analyzed at the phylum, class, order, family, genus, and species levels.

Rarefaction curves were generated by treating all experimental samples as a whole, and randomly subsampling a certain number of individuals to count the microbial species they represented. The number of individuals sampled was plotted on the x-axis, and the number of observed species on the y-axis. A smoothly plateauing curve indicated that the sequencing depth was sufficient.

Species accumulation curves were used to evaluate species richness. A curve trending linearly suggested insufficient sampling, whereas a curve reaching a plateau indicated adequate sampling effort.

### Taxonomic composition analysis

Community composition bar plots were generated based on taxonomic classification results, allowing for intuitive visualization of community structures and changes across samples at different taxonomic levels. These plots could only indicate relative increases or decreases in abundance, but did not provide statistical significance. Krona analysis was performed using the Krona software to visualize taxonomic information of samples across groups at different classification levels. In the resulting circular plots, concentric layers from the center to the periphery represented different taxonomic ranks, and the size of each sector corresponded to the relative proportion of OTUs annotated at that level.

### Alpha diversity analysis

Alpha diversity refers to the within-sample diversity, reflecting species richness and evenness. A series of indices, including Chao, ACE, Shannon, Simpson, and PD-whole-tree, were calculated. Rarefaction curves of each index were plotted using the Mothur software to evaluate sequencing depth and sample diversity.

### Beta diversity analysis

Beta diversity refers to the diversity between samples. Distances between samples were calculated using the Bray–Curtis algorithm to generate a distance matrix. This matrix was then analyzed with principal coordinates analysis (PCoA) and non-metric multidimensional scaling (NMDS). The degree of clustering or separation of points in the two-dimensional plots reflected differences in microbial community composition among samples. A shorter distance between points indicated smaller differences in species diversity between the corresponding samples.

### Differential analysis of species between groups

Metastats was used to perform significance testing of species differences between two or more groups at various taxonomic levels. Raw Metastats *P* values were adjusted for multiple comparisons at each taxonomic rank using the Benjamini–Hochberg false-discovery-rate (BH-FDR) procedure, and only taxa with adjusted $q < 0.05$ are reported as statistically significant in the main text. Taxa that were significant at unadjusted $P < 0.05$ but did not survive FDR correction are reported as exploratory in the Supplementary Materials.

LEfSe (Linear discriminant analysis Effect Size) analysis was applied to identify biomarkers with significantly different abundances among two or more groups and their associated categories. The default logarithmic LDA threshold of $>2.0$ was retained as a biologically motivated effect-size filter. Taxa with the greatest influence on the differences were determined and visualized through LDA score plots, LEfSe cladograms, and bar plots.

### Compositional sensitivity analysis (ANCOM and ANCOM-BC)

To account for the compositional nature of microbiome count data, all primary differential-abundance comparisons were independently validated using both ANCOM (Analysis of Composition of Microbiomes) [9] and ANCOM-BC (with Bias Correction) [10] as implemented in the scikit-bio library (v. 0.7). ANCOM employs pairwise log-ratio tests across all taxa and reports a W-statistic; ANCOM-BC uses a linear regression framework on log-transformed counts with sample-specific bias correction and reports log$_2$ fold-changes with BH-FDR-adjusted *q*-values. Taxa were considered robust if they reached significance in ANCOM-BC ($q<0.05$); these robust taxa form the basis of the conclusions in the main text.

### Statistical methods

Differences in alpha diversity were assessed using the non-parametric Kruskal–Wallis test, with $p < 0.05$ considered statistically significant. Differences in beta diversity were evaluated using Bray–Curtis distance algorithms combined with NMDS and PCoA analyses, and formally tested with PERMANOVA (999 permutations); distance values ranged from 0 to 1, with larger values indicating greater differences between samples. Differential species analysis between groups was conducted using Metastats and LEfSe with BH-FDR correction as described above, and independently validated by ANCOM-BC; an FDR-adjusted $q < 0.05$ was considered statistically significant for all differential-abundance results reported in the main text.

## Experiment results

### Grouping results

1. RE group: 7 cases; NRE group: 23 cases.
2. B1 group: 7 cases; B2 group: 5 cases; B3 group: 18 cases.
3. NHS3_1 group: 8 cases; NHS3_2 group: 13 cases; NHS3_3 group: 9 cases.

### Sequencing results

**Fig. 1:**
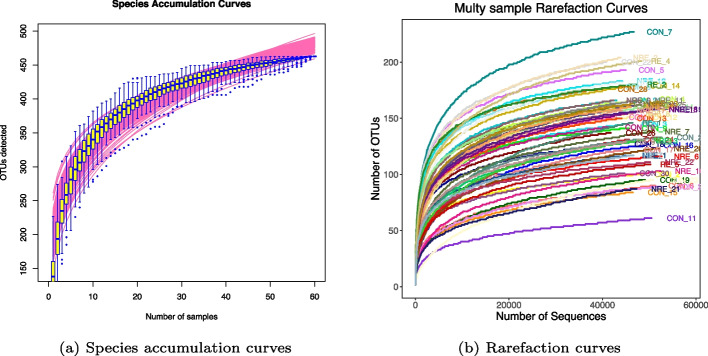
Species accumulation curves (**a**) and rarefaction curves (**b**) based on random subsampling of sequencing data from each sample. Both curves plateaued, indicating sufficient sequencing depth and adequate representation of species diversity

A total of 3,029,486 valid sequences with barcode tags were obtained from the fecal samples of 60 subjects. After quality filtering and sequence merging, 28,822,225 optimized sequences were generated. Species accumulation curves (Fig. 1a) and rarefaction curves (Fig. 1b), plotted based on random subsampling of sequences from each sample, showed that the curves approached a plateau, indicating sufficient sequencing depth. At a 97% similarity threshold, the sequencing data were adequate to capture the species diversity within the samples.

### Diversity results

#### Alpha diversity analysis

**Fig. 2:**
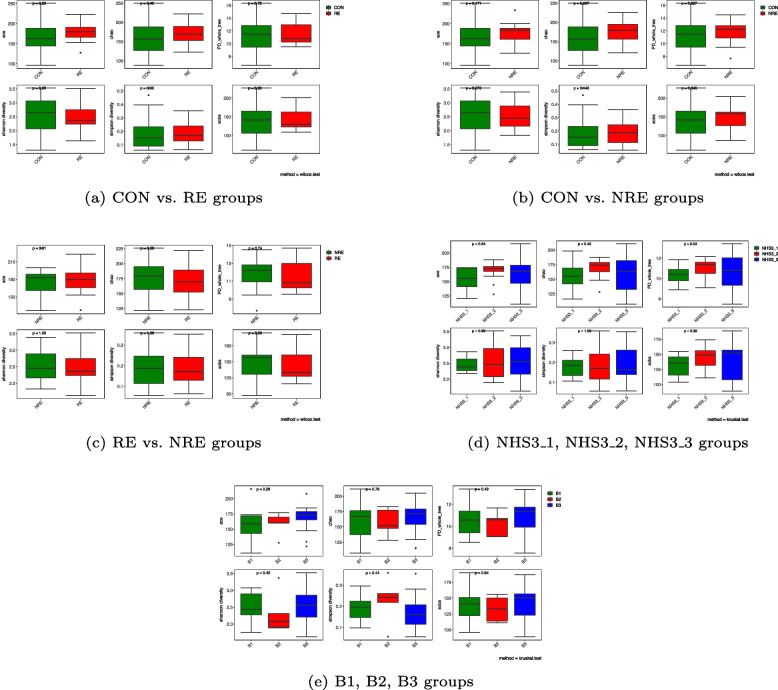
Alpha diversity comparisons of gut microbiota: **a** CON vs. RE groups; **b** CON vs. NRE groups; **c** RE vs. NRE groups; **d** NHS3_1, NHS3_2, NHS3_3 groups; **e** B1, B2, B3 groups

No statistically significant differences in gut microbiota alpha diversity were observed between the CON and RE groups (Fig. 2a), the CON and NRE groups (Fig. 2b), the RE and NRE groups (Fig. 2c), among the NHS3_1, NHS3_2, and NHS3_3 groups (Fig. 2d), or among the B1, B2, and B3 groups (Fig. 2e) ($P> 0.05$).

#### Beta diversity analysis results

**Fig. 3:**
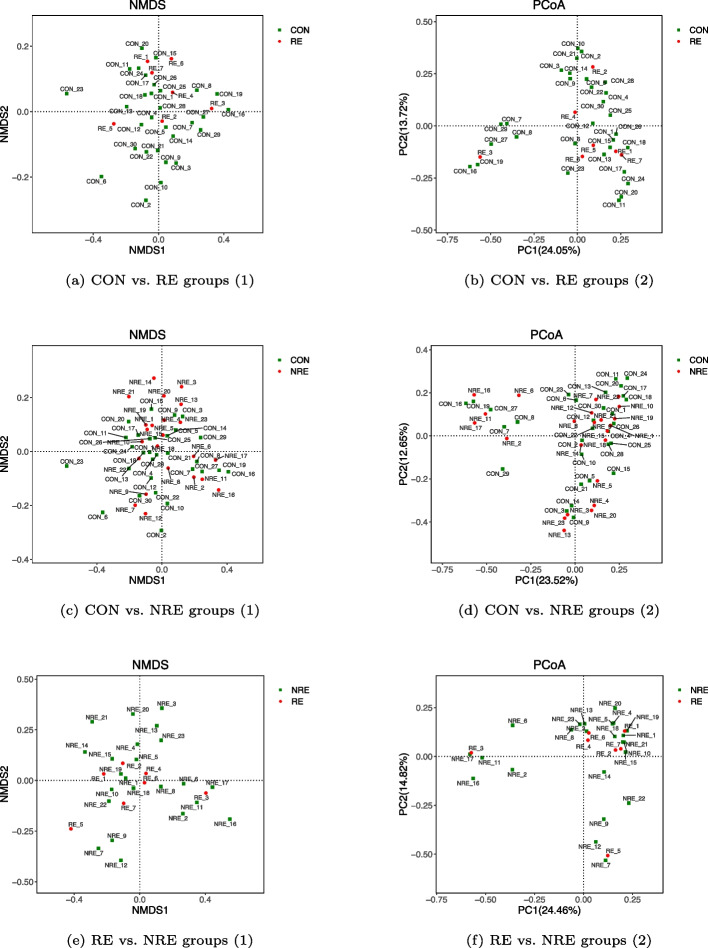
Beta diversity comparisons of gut microbiota (Part 1): **a**-**b** CON vs. RE groups; **c**-**d** CON vs. NRE groups; **e**-**f** RE vs. NRE groups

**Fig. 4:**
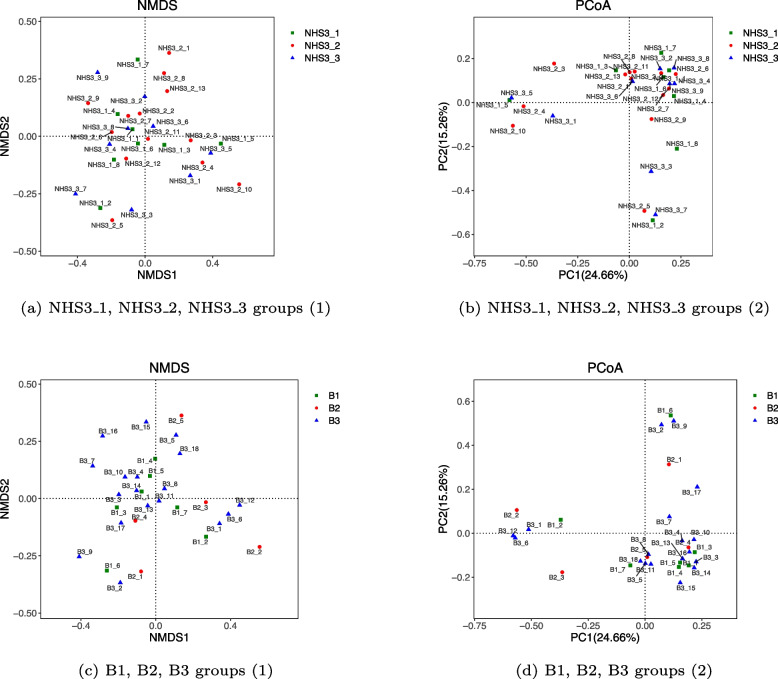
Beta diversity comparisons of gut microbiota (Part 2): **a**-**b** NHS3_1, NHS3_2, NHS3_3 groups; **c**-**d** B1, B2, B3 groups

No statistically significant differences in gut microbiota beta diversity were observed between the CON and RE groups (Fig. 3a-b), the CON and NRE groups (Fig. 3c-d), the RE and NRE groups (Fig. 3e-f), among the NHS3_1, NHS3_2, and NHS3_3 groups (Fig. 4a-b), or among the B1, B2, and B3 groups (Fig. 4c-d) ($P> 0.05$).

#### Analysis of intergroup differences

Differential abundance analysis was performed using Metastats with Benjamini–Hochberg FDR correction and independently validated with ANCOM-BC. Unadjusted Metastats *P* values and full taxon lists are provided in Tables 1, 2, 3, 4, 5 and 6 and Supplementary Tables S1–S8; only key findings are summarized below. It is important to note that a substantial proportion of taxa reaching unadjusted $P < 0.05$ did not survive FDR correction, and the results discussed here emphasize ANCOM-BC-validated taxa where available.

**Differences in microbial taxa between the CON and RE groups**. Metastats identified 21 taxa at unadjusted $P < 0.05$ across family, genus, and species levels (Table 1); however, none survived BH-FDR correction at $q < 0.05$ (Supplementary Table S1). By LEfSe, *Bacteroides plebeius* and *Megamonas* contributed most to group separation (Supplementary Figs. S3–S6). ANCOM-BC independently identified significant depletion of *Bacteroides plebeius* ($\log _2\textrm{FC} = -2.80$, $q = 0.002$) and *Coprococcus comes* ($\log _2\textrm{FC} = -2.05$, $q = 0.002$) in the RE group, representing the most robust species-level findings in this study (Supplementary Table S4).

**Table 1:** Changes in gut microbiota abundance at different taxonomic levels in the RE group compared with the CON group. $^\dagger$Unadjusted Metastats *P* values; Benjamini–Hochberg FDR-adjusted *q*-values are reported in Supplementary Table S1. ANCOM-BC-validated taxa are indicated in the Discussion

Taxonomic level	Differential taxa	Abundance change	*P* value$^\dagger$
Family	*Propionibacteriaceae*	Significantly increased	$P < 0.01$
	*Microbacteriaceae*	Increased	$P < 0.05$
	*Staphylococcaceae*	Increased	$P < 0.05$
	*Moraxellaceae*	Significantly decreased	$P < 0.01$
	*Planococcaceae*	Significantly decreased	$P < 0.01$
Genus	*Propionibacterium*	Significantly increased	$P < 0.01$
	*Johnsonella*	Significantly increased	$P < 0.01$
	*Microcella*	Increased	$P < 0.05$
	*Staphylococcus*	Increased	$P < 0.05$
	*Acinetobacter*	Significantly decreased	$P < 0.01$
	*Dialister*	Significantly decreased	$P < 0.01$
	*Lysinibacillus*	Significantly decreased	$P < 0.01$
	*Dielma*	Decreased	$P < 0.05$
Species	*Campylobacter_concisus*	Significantly increased	$P < 0.01$
	*Microcella_alkaliphila*	Increased	$P < 0.05$
	*Ruminococcaceae_bacterium*	Increased	$P < 0.05$
	*Campylobacter_ureolyticus*	Increased	$P < 0.05$
	*Eubacterium_ramulus*	Decreased	$P < 0.05$
	*Bacteroides_plebeius*	Decreased	$P < 0.05$
	*Coprococcus_comes*	Significantly decreased	$P < 0.01$
	*gut_metagenome*	Decreased	$P < 0.05$

**Table 2:** Changes in gut microbiota abundance at different taxonomic levels in the NRE group compared with the CON group. $^\dagger$Unadjusted Metastats *P* values; FDR-adjusted *q*-values in Supplementary Table S2

Taxonomic level	Differential taxa	Abundance change	*P* value$^\dagger$
Phylum	*Proteobacteria*	Significantly decreased	$P < 0.01$
Family	*Moraxellaceae*	Significantly decreased	$P < 0.01$
	*Planococcaceae*	Significantly decreased	$P < 0.01$
	*Neisseriaceae*	Significantly decreased	$P < 0.01$
	*Enterobacteriaceae*	Decreased	$P < 0.05$
	*Leptotrichiaceae*	Increased	$P < 0.05$
	*Eubacteriaceae*	Increased	$P < 0.05$
Genus	*Acinetobacter*	Significantly decreased	$P < 0.01$
	*Lysinibacillus*	Significantly decreased	$P < 0.01$
	*Eikenella*	Significantly decreased	$P < 0.01$
	*Intestinibacter*	Decreased	$P < 0.05$
	*Rikenella*	Significantly decreased	$P < 0.01$
	*Candidatus_Soleaferrea*	Significantly decreased	$P < 0.01$
	*Acidaminococcus*	Significantly decreased	$P < 0.01$
	*Shuttleworthia*	Significantly increased	$P < 0.001$
	*Sneathia*	Significantly increased	$P < 0.001$
	*Mogibacterium*	Significantly increased	$P < 0.001$
	*Murdochiella*	Significantly increased	$P < 0.001$
	*Peptostreptococcus*	Significantly increased	$P < 0.001$
	*Faecalitalea*	Significantly increased	$P < 0.001$
	*Eubacterium*	Significantly increased	$P < 0.001$
Species	*Bacteroidetes_bacterium*	Significantly decreased	$P < 0.001$
	*Intestinibacter_bartlettii*	Decreased	$P < 0.05$
	*Acidaminococcus_fermentans*	Decreased	$P < 0.05$
	*Escherichia_coli*	Decreased	$P < 0.05$
	*Collinsella_tanakaei*	Significantly increased	$P < 0.001$
	*Eubacterium_sp._cL-10-1-3*	Increased	$P < 0.05$
	*Ruminococcus_sp._WAL_17306*	Increased	$P < 0.05$
	*Campylobacter_hominis*	Increased	$P < 0.05$
	*Mogibacterium_diversum*	Increased	$P < 0.05$
	*Bacteroides_coagulans*	Increased	$P < 0.05$
	*Eubacterium_limosum*	Increased	$P < 0.05$

**Fig. 5:**
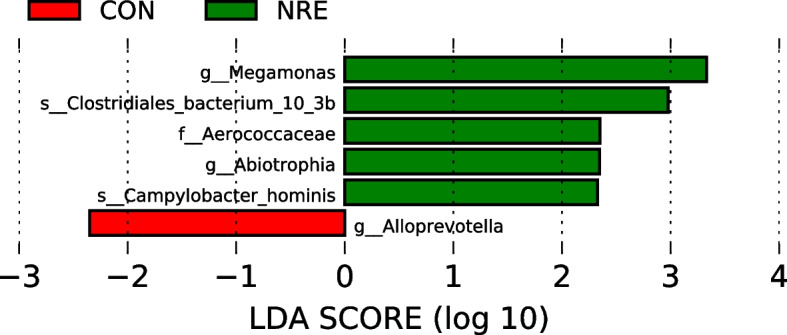
LEfSe LDA score plot showing differences in gut microbiota between CON and NRE groups

**Differences in microbial taxa between the CON and NRE groups**. Metastats identified 31 taxa at unadjusted $P < 0.05$ (Table 2); after FDR correction, 10 taxa remained significant at $q < 0.05$, including *Moraxellaceae*, *Planococcaceae*, and *Neisseriaceae* at the family level and *Acinetobacter*, *Lysinibacillus*, *Eikenella*, *Shuttleworthia*, and *Sneathia* at the genus level (Supplementary Table S2). By LEfSe, *Alloprevotella* (CON) and *Megamonas* (NRE) contributed most to group separation (Fig. 5; Supplementary Figs. S7–S9). However, ANCOM-BC did not confirm any of these taxa at $q < 0.05$, suggesting that these associations may be sensitive to the analytical framework used.

**Differences in microbial taxa between the RE and NRE groups**. Metastats identified 30 taxa at unadjusted $P < 0.05$ (Table 3); none survived FDR correction (Supplementary Table S3). ANCOM-BC identified depletion of *Synergistetes* (phylum, $q = 0.008$) and species-level depletion of *Bacteroides plebeius*, *Coprococcus comes*, and *Sutterella wadsworthensis* in the RE relative to NRE group, consistent with the CON-vs-RE findings (Supplementary Table S4; Supplementary Figs. S10–S13).

**Table 3:** Changes in gut microbiota abundance at different taxonomic levels in the RE group compared with the NRE group. $^\dagger$Unadjusted Metastats *P* values; FDR-adjusted *q*-values in Supplementary Table S3

Taxonomic level	Differential taxa	Abundance change	*P* value$^\dagger$
Phylum	*Tenericutes*	Significantly decreased	$P < 0.01$
	*Synergistetes*	Decreased	$P < 0.05$
Family	*Staphylococcaceae*	Increased	$P < 0.05$
	*Propionibacteriaceae*	Increased	$P < 0.05$
	*Microbacteriaceae*	Increased	$P < 0.05$
Genus	*Dialister*	Significantly decreased	$P < 0.01$
	*Pseudobutyrivibrio*	Significantly decreased	$P < 0.01$
	*Mitsuokella*	Decreased	$P < 0.05$
	*Pyramidobacter*	Decreased	$P < 0.05$
	*Senegalimassilia*	Decreased	$P < 0.05$
	*Cloacibacillus*	Decreased	$P < 0.05$
	*Ruminococcus*	Decreased	$P < 0.05$
	*Klebsiella*	Decreased	$P < 0.05$
	*Staphylococcus*	Increased	$P < 0.05$
	*Propionibacterium*	Increased	$P < 0.05$
	*Stomatobaculum*	Increased	$P < 0.05$
	*Cloacibacillus*	Increased	$P < 0.05$
	*Microcella*	Increased	$P < 0.05$
Species	*Pyramidobacter_piscolens*	Decreased	$P < 0.05$
	*Bacteroides_plebeius*	Decreased	$P < 0.05$
	*Sutterella_wadsworthensis*	Decreased	$P < 0.05$
	*Lactobacillus_prophage_Lj928*	Decreased	$P < 0.05$
	*Bacteroides_coagulans*	Decreased	$P < 0.05$
	*Prevotella_disiens*	Decreased	$P < 0.05$
	*Cloacibacillus_evryensis*	Decreased	$P < 0.05$
	*Ruminococcus_sp._WAL_17306*	Decreased	$P < 0.05$
	*Bacteroides_coprocola*	Decreased	$P < 0.05$
	*Coprococcus_comes*	Significantly decreased	$P < 0.01$
	*Campylobacter_concisus*	Significantly increased	$P < 0.01$
	*Microcella_alkaliphila*	Increased	$P < 0.05$

**Differences in microbial taxa among the NHS3_1, NHS3_2, and NHS3_3 seizure-severity groups**. At unadjusted $P < 0.05$, Metastats suggested progressive changes with increasing seizure severity, including depletion of *Eubacteriaceae*/*Eubacterium* and enrichment of *Cloacibacillus* (Supplementary Figs. S14–S15, Supplementary Table S8). However, after FDR correction, these genus- and family-level trends did not reach $q < 0.05$. ANCOM-BC identified only *Parasutterella excrementihominis* (species) as significantly enriched in NHS3_2 vs. NHS3_1 ($q = 0.012$; Supplementary Table S4). LEfSe analysis is shown in Supplementary Figs. S16–S18. These findings suggest that seizure-severity-associated microbial patterns, while present at the exploratory level, are not robust to multiple-testing correction in this sample.

**Fig. 6:**
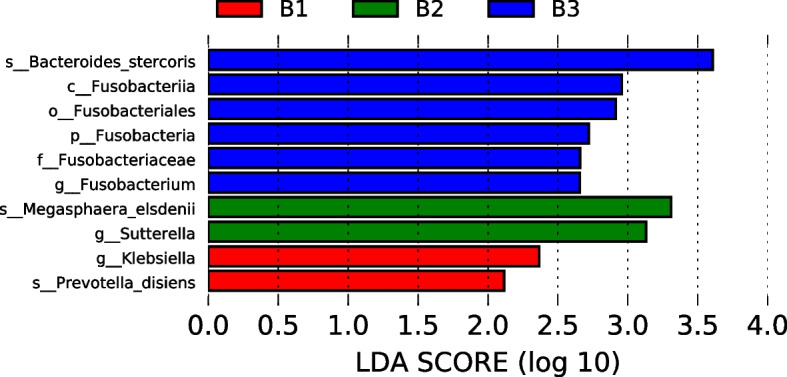
LEfSe LDA score plot showing differences in gut microbiota among B1, B2, and B3 groups

**Differences in microbial taxa among the B1, B2, and B3 psychiatric-comorbidity groups**. Metastats identified multiple taxa at unadjusted $P < 0.05$ across the B1 (no comorbidity), B2 (depression), and B3 (anxiety + depression) groups (Tables 4, 5 and 6); however, no taxa survived BH-FDR correction in any pairwise comparison (Supplementary Tables S5–S7). By LEfSe, *Klebsiella* (B1), *Megasphaera elsdenii* (B2), and *Bacteroides stercoris* (B3) contributed most to group separation (Fig. 6; Supplementary Figs. S19–S23).

Importantly, ANCOM-BC identified several robust signals in these subgroups despite the small sample sizes. *Ruminococcus* was significantly depleted in the B2 group relative to B1 ($\log _2\textrm{FC} = -4.44$, $q = 0.0003$), further supported by ANCOM ($W = 15$). *Bilophila* was enriched in B3 relative to B1 ($q = 0.033$). The B3-vs-B2 contrast revealed strong enrichment of *Bacteroides stercoris* (ANCOM-BC $q = 8 \times 10^{-6}$; ANCOM $W = 75$) and depletion of *Sutterella* ($q = 6 \times 10^{-5}$), as well as enrichment of *Fusobacteria* at the phylum level ($q = 0.0006$). These ANCOM-BC-validated findings suggest partially distinct microbial signatures for depression-only versus anxiety-plus-depression comorbidity in TLE patients, although the small subgroup sizes (B1 $n = 7$; B2 $n = 5$; B3 $n = 18$) warrant cautious interpretation.

**Table 4:** Changes in gut microbiota abundance at different taxonomic levels in the B2 group compared with the B1 group. $^\dagger$Unadjusted Metastats *P* values; FDR-adjusted *q*-values in Supplementary Table S5. ANCOM-BC-validated taxa are discussed in the text

Taxonomic level	Differential taxa	Abundance change	*P* value$^\dagger$
Phylum	*Proteobacteria*	Increased	$P < 0.05$
Family	*Pasteurellaceae*	Increased	$P < 0.05$
	*Ruminococcaceae*	Increased	$P < 0.05$
	*Alcaligenaceae*	Increased	$P < 0.05$
Genus	*Ruminococcus*	Significantly decreased	$P < 0.01$
	*Faecalibacterium*	Decreased	$P < 0.05$
	*Haemophilus*	Increased	$P < 0.05$

**Table 5:** Changes in gut microbiota abundance at different taxonomic levels in the B3 group compared with the B1 group. $^\dagger$Unadjusted Metastats *P* values; FDR-adjusted *q*-values in Supplementary Table S6

Taxonomic level	Differential taxa	Abundance change	*P* value$^\dagger$
Phylum	*Campylobacteraceae*	Increased	$P < 0.05$
Genus	*Bilophila*	Significantly increased	$P < 0.01$
	*Cloacibacillus*	Significantly increased	$P < 0.01$
	*Intestinibacter*	Increased	$P < 0.05$
	*Erysipelatoclostridium*	Increased	$P < 0.05$
	*Parasutterella*	Increased	$P < 0.05$
	*Peptostreptococcus*	Increased	$P < 0.05$
	*Campylobacter*	Increased	$P < 0.05$
	*Johnsonella*	Increased	$P < 0.05$

**Table 6:** Changes in gut microbiota abundance at different taxonomic levels in the B3 group compared with the B2 group. $^\dagger$Unadjusted Metastats *P* values; FDR-adjusted *q*-values in Supplementary Table S7

Taxonomic level	Differential taxa	Abundance change	*P* value$^\dagger$
Family	*Acidaminococcaceae*	Increased	$P < 0.05$
Genus	*Phascolarctobacterium*	Increased	$P < 0.05$
	*Ruminococcus*	Increased	$P < 0.05$
	*Desulfovibrio*	Increased	$P < 0.05$
	*Cloacibacillus*	Increased	$P < 0.05$
	*Adlercreutzia*	Increased	$P < 0.05$
	*Sutterella*	Decreased	$P < 0.05$

## Discussion

The principal observation of this study is that, in temporal-lobe epilepsy (TLE), it is the composition of specific taxa—not overall $\alpha$- or $\beta$-diversity—that distinguishes patients from family-matched controls. The absence of significant community-level diversity differences alongside taxon-level differences is not intrinsically contradictory: global diversity indices summarize broad ecological structure, whereas differential-abundance testing detects localized shifts in particular lineages. Similar discordance has been reported in other neuropsychiatric microbiome studies [11, 12]. Importantly, many of the taxa identified as differentially abundant using Metastats at unadjusted $P < 0.05$ did not survive Benjamini–Hochberg FDR correction, underscoring the need for rigorous multiple-testing control in high-dimensional microbiome analyses. To further address the compositional nature of 16S rRNA data, we independently validated all comparisons using ANCOM-BC; only taxa reaching $q < 0.05$ under ANCOM-BC are discussed as primary findings below. The remaining Metastats-only taxa are reported in the Supplementary Materials as exploratory.

Among the comparisons between drug-resistant epilepsy (RE) and healthy controls, ANCOM-BC identified robust species-level signatures (Supplementary Fig. S1). *Bacteroides plebeius* ($\log _2\textrm{FC} = -2.80$, $q = 0.002$) and *Coprococcus comes* ($\log _2\textrm{FC} = -2.05$, $q = 0.002$) were both significantly depleted in RE patients. *Coprococcus* is a well-characterized butyrate-producing genus; its depletion is consistent with reduced short-chain fatty acid (SCFA) availability, which has been linked to impaired intestinal barrier integrity and increased systemic inflammation [13]. *Bacteroides plebeius*, a polysaccharide-degrading species, has been associated with dietary fiber metabolism and maintenance of colonic homeostasis. Their concurrent depletion in drug-resistant patients—but not in the non-drug-resistant (NRE) group—suggests a possible microbial signature of treatment-refractory disease, although this comparison ($n = 7$ for RE) remains underpowered and requires confirmation in larger cohorts. These RE-specific findings also emerged in the RE-vs-NRE contrast, alongside depletion of *Sutterella wadsworthensis*, indicating that the drug-resistant subgroup harbors a distinct microbial profile relative to both controls and non-resistant patients. Consistent with prior reports [8], we observed altered gut microbiota in drug-resistant epilepsy; however, the specific taxa differ from those previously highlighted (e.g., *Bifidobacterium* and *Lactobacillus*), likely reflecting differences in cohort characteristics, geography, dietary habits, and analytical methods.

Subgroup analyses by psychiatric comorbidity, although involving small sample sizes (B1 $n = 7$; B2 $n = 5$; B3 $n = 18$), revealed several ANCOM-BC-significant findings. The most robust signal was a pronounced depletion of *Ruminococcus* in the depression-only subgroup (B2 vs. B1: $\log _2\textrm{FC} = -4.44$, $q = 0.0003$, further supported by ANCOM $W = 15$). *Ruminococcus* spp. are major fiber-fermenting commensals involved in SCFA production and mucin degradation; their reduction has been reported in major depressive disorder outside the epilepsy context [14]. The comorbid anxiety-plus-depression subgroup (B3) showed enrichment of *Bilophila* relative to patients without psychiatric comorbidity (B3 vs. B1: $q = 0.033$). *Bilophila wadsworthia* is a sulfite-reducing, pro-inflammatory organism whose expansion has been associated with high-fat diets and intestinal inflammation. The B3-vs-B2 contrast further revealed strong enrichment of *Bacteroides stercoris* (ANCOM-BC $q = 8 \times 10^{-6}$; ANCOM $W = 75$, the highest observed in this study) and depletion of *Sutterella* ($q = 6 \times 10^{-5}$). These between-subgroup differences suggest that comorbid anxiety-plus-depression and depression alone may involve partially distinct microbial signatures, a hypothesis that warrants testing in adequately powered cohorts.

In contrast, the primary TLE-vs-CON comparison (all 30 patients vs. 30 controls) and the NRE-vs-CON comparison did not yield ANCOM-BC-robust findings, despite multiple taxa reaching nominal significance in Metastats. Similarly, seizure-severity stratification by NHS3 scores produced only a single ANCOM-BC-significant species (*Parasutterella excrementihominis*, NHS3-2 vs. NHS3-1, $q = 0.012$), insufficient to support the progressive-gradient narrative suggested by exploratory Metastats results. This pattern—a rich Metastats signal that largely dissolves under FDR correction and compositional re-analysis (Supplementary Fig. S2)—highlights that a substantial proportion of previously reported taxon-level associations in small epilepsy microbiome studies may represent false positives, a concern explicitly raised in recent reviews [11].

This study has several important limitations. (i) The overall sample is modest ($n = 30$ per arm) and some clinically defined subgroups (drug-resistant, $n = 7$; B2 depression only, $n = 5$) are underpowered for formal hypothesis testing; subgroup findings are therefore exploratory and hypothesis-generating. (ii) The cross-sectional design precludes any causal inference: all associations reported here are correlative, and directionality (microbial shift as driver vs. consequence of disease or treatment) cannot be established. (iii) 16S rRNA sequencing provides taxonomic resolution but limited functional information; inferences about SCFA production or inflammatory potential are based on known taxon biology rather than direct metabolomic measurement. (iv) Residual confounding by antiseizure medication type, duration, and unmeasured dietary variation cannot be fully excluded, although family-matched controls sharing the same household diet partially mitigate dietary confounders [15]. (v) Our bioinformatic pipeline used OTU clustering at 97% similarity rather than contemporary ASV-based methods (DADA2, Deblur); future work should employ ASV-level resolution. (vi) Single-center recruitment from a referral neurology service limits generalizability.

In summary, this study identifies a small set of candidate microbial associations with drug-resistant TLE and psychiatric comorbidity, validated by compositional-aware statistical methods. Key candidate taxa—*Bacteroides plebeius* and *Coprococcus comes* (drug resistance), *Ruminococcus* (depression comorbidity), *Bilophila* and *Bacteroides stercoris* (anxiety-plus-depression)—merit prioritized validation. Future work should (a) assemble larger and, ideally, drug-naïve or newly diagnosed cohorts to separate disease from treatment effects; (b) complement 16S rRNA profiling with shotgun metagenomics and targeted metabolomics (SCFAs, bile acids, neuroactive metabolites) for direct functional inference; (c) pursue longitudinal sampling to clarify temporal ordering relative to seizure events and psychiatric symptomatology; and (d) test mechanistic hypotheses in gnotobiotic animal models. Microbiota-targeted interventions should be evaluated only as candidate strategies for future controlled trials rather than as immediate clinical recommendations.

## Conclusions

This study reports that patients with temporal lobe epilepsy exhibit gut microbiota profiles associated with their clinical status compared with family-matched healthy controls, characterized by specific taxon-level changes rather than overall diversity differences. After rigorous Benjamini–Hochberg FDR correction and independent validation with ANCOM-BC, the following exploratory findings emerged: (1) drug-resistant epilepsy was associated with significant depletion of *Bacteroides plebeius* and *Coprococcus comes* at the species level; (2) comorbid depression was associated with pronounced reduction of *Ruminococcus*; and (3) the comorbid anxiety-plus-depression subgroup was distinguished from the depression-only subgroup by enrichment of *Bacteroides stercoris* (confirmed by both ANCOM and ANCOM-BC) and *Bilophila*. A substantial proportion of taxa identified at unadjusted $P < 0.05$ in standard Metastats analysis did not survive FDR correction or compositional re-analysis, highlighting the importance of stringent statistical control in microbiome studies. Given the cross-sectional design, modest sample size, and underpowered subgroups, these findings should be interpreted as hypothesis-generating associations rather than causal or diagnostic claims. They identify a small set of candidate microbial markers warranting prioritized validation in larger, longitudinal cohorts, combined with shotgun metagenomics, targeted metabolomics, and mechanistic studies in animal models, before any microbiota-targeted therapeutic strategy could be considered.

## Supplementary Information

Supplementary Material 1.

## Data Availability

The 16S rRNA gene representative OTU sequences have been deposited in NCBI GenBank under accession numbers PZ117880–PZ118329. The processed datasets (OTU abundance tables, alpha diversity indices, LEfSe results, and differential analysis data) are available in the Figshare repository (https://doi.org/10.6084/m9.figshare.31564168). Raw sequencing data are available from the corresponding author upon reasonable request.
