# Supplementary material for: Gut microbiota profiles associated with temporal lobe epilepsy and psychiatric comorbidities: a family-matched case–control 16S rRNA study
Source: BMC Neurol. 2026 May 12;26:433. doi: 10.1186/s12883-026-04958-7 (PMC13335196; doi:10.1186/s12883-026-04958-7)
Supplement: Supplementary file 1 — Supplementary Material 1. [file 12883_2026_4958_MOESM1_ESM.pdf]

Supplementary Materials

Gut Microbiota Profiles Associated with Temporal Lobe Epilepsy and Psychiatric Comorbidities

Contents

1 Supplementary Tables **3**

2 Supplementary Figures **11**

Overview

This document provides the full supplementary materials supporting the revised manuscript. It includes:

- **Supplementary Tables S1–S8**: Complete Benjamini–Hochberg FDR-corrected Metastats differential-abundance results and the full ANCOM / ANCOM-BC output for every contrast and taxonomic rank. Companion machine-readable TSV files accompany this PDF.
- **Supplementary Figures S1–S2**: Visual summaries of the compositional sensitivity analysis (ANCOM-BC forest plot) and the impact of FDR correction on Metastats results.
- **Supplementary Figures S3–S23**: The 21 Metastats bar plots, LEfSe LDA plots, LEfSe cladograms, and LEfSe bar plots that were relocated from the main text to supplementary in response to Reviewer 1's "Figure Overload" comment.

1 Supplementary Tables

Supplementary Table 1: FDR-corrected Metastats: CON vs. RE. Unadjusted $P < 0.05$ taxa; bold q indicates FDR-significant at $q < 0.05$.

**Level**	**Taxon**	P **value**	**BH-FDR** q
family	*Propionibacteriaceae*	0.00141	0.0704
family	*Moraxellaceae*	0.00353	0.0883
family	*Planococcaceae*	0.00861	0.144
family	*Microbacteriaceae*	0.0259	0.299
family	*Staphylococcaceae*	0.0299	0.299
genus	*Propionibacterium*	0.00141	0.161
genus	*Acinetobacter*	0.00353	0.161
genus	*Johnsonella*	0.00403	0.161
genus	*Dialister*	0.00681	0.204
genus	*Lysinibacillus*	0.00861	0.207
genus	*Dielma*	0.0165	0.33
genus	*Microcella*	0.0259	0.444
genus	*Staphylococcus*	0.0299	0.448
order	*Propionibacteriales*	0.00141	**0.0408**
otu	*OTU483*	0.00141	0.275
otu	*OTU223*	0.00347	0.275
otu	*OTU327*	0.00353	0.275
otu	*OTU372*	0.00403	0.275
otu	*OTU22*	0.00412	0.275
otu	*OTU278*	0.00546	0.275
otu	*OTU189*	0.00595	0.275
otu	*OTU460*	0.00595	0.275
otu	*OTU472*	0.00727	0.275
otu	*OTU75*	0.00744	0.275
otu	*OTU336*	0.0078	0.275
otu	*OTU342*	0.00861	0.275
otu	*OTU359*	0.00861	0.275
otu	*OTU264*	0.0089	0.275
otu	*OTU74*	0.00932	0.275
otu	*OTU275*	0.0104	0.287
otu	*OTU3*	0.0122	0.317
otu	*OTU293*	0.0136	0.33
otu	*OTU442*	0.0142	0.33
otu	*OTU72*	0.0176	0.373
otu	*OTU78*	0.0177	0.373
otu	*OTU257*	0.0226	0.408
otu	*OTU300*	0.0226	0.408
otu	*OTU324*	0.0226	0.408
otu	*OTU160*	0.0246	0.408
otu	*OTU402*	0.0249	0.408

Showing top 40 taxa by BH-FDR q-value; full results in companion TSV file.

Supplementary Table 2: FDR-corrected Metastats: CON vs. NRE. Unadjusted $P < 0.05$ taxa; bold q indicates FDR-significant.

**Level**	**Taxon**	P **value**	**BH-FDR** q
class	*Gammaproteobacteria*	0.047	0.713
family	*Moraxellaceae*	0	**0**
family	*Planococcaceae*	1e-06	**2.5e-05**
family	*Neisseriaceae*	8.5e-05	**0.00142**
family	*Enterobacteriaceae*	0.021	0.262
family	*Leptotrichiaceae*	0.0344	0.344
family	*Eubacteriaceae*	0.0493	0.411
genus	*Acinetobacter*	0	**0**
genus	*Lysinibacillus*	1e-06	**6.05e-05**
genus	*Eikenella*	8.5e-05	**0.00343**
genus	*Shuttleworthia*	0.000645	**0.0195**
genus	*Sneathia*	0.0009	**0.0218**
genus	*Intestinibacter*	0.011	0.222
genus	*Mogibacterium*	0.0193	0.276
genus	*Rikenella*	0.0201	0.276
genus	*Murdochiella*	0.0205	0.276
genus	*Candidatus_Soleaferrea*	0.0231	0.28
genus	*Peptostreptococcus*	0.0272	0.3
genus	*Faecalitalea*	0.04	0.381
genus	*Acidaminococcus*	0.041	0.381
genus	*Eubacterium*	0.0493	0.419
order	*Neisseriales*	8.5e-05	**0.00247**
order	*Enterobacteriales*	0.029	0.297
order	*Rickettsiales*	0.0307	0.297
otu	*OTU299*	0	**0**
otu	*OTU452*	0	**0**
otu	*OTU342*	1e-06	**0.000114**
otu	*OTU359*	1e-06	**0.000114**
otu	*OTU300*	6e-06	**0.000391**
otu	*OTU324*	6e-06	**0.000391**
otu	*OTU361*	6e-06	**0.000391**
otu	*OTU333*	1e-05	**0.00057**
otu	*OTU250*	5.5e-05	**0.00255**
otu	*OTU433*	5.6e-05	**0.00255**
otu	*OTU318*	8.5e-05	**0.00352**
otu	*OTU389*	0.000196	**0.00745**
otu	*OTU328*	0.000376	**0.0132**
otu	*OTU289*	0.00053	**0.0173**
otu	*OTU330*	0.000645	**0.0184**
otu	*OTU413*	0.000645	**0.0184**

Showing top 40 taxa by BH-FDR q-value; full results in companion TSV file.

Supplementary Table 3: FDR-corrected Metastats: RE vs. NRE. No taxa survived FDR correction at any taxonomic rank.

**Level**	**Taxon**	P **value**	**BH-FDR** q
class	*Mollicutes*	0.00715	0.129
family	*Staphylococcaceae*	0.0119	0.286
family	*Propionibacteriaceae*	0.0119	0.286
family	*Microbacteriaceae*	0.0394	0.578
genus	*Dialister*	0.00117	0.134
genus	*Pseudobutyrivibrio*	0.00443	0.181
genus	*Mitsuokella*	0.0117	0.181
genus	*Pyramidobacter*	0.0117	0.181
genus	*Senegalimassilia*	0.0117	0.181
genus	*Staphylococcus*	0.0119	0.181
genus	*Propionibacterium*	0.0119	0.181
genus	*Stomatobaculum*	0.0126	0.181
genus	*Cloacibacillus*	0.0322	0.412
genus	*Ruminococcus*	0.0372	0.412
genus	*Microcella*	0.0394	0.412
genus	*Klebsiella*	0.0435	0.417
order	*Propionibacteriales*	0.0119	0.346
otu	*OTU443*	0.000681	0.247
otu	*OTU22*	0.00117	0.247
otu	*OTU75*	0.00229	0.294
otu	*OTU460*	0.00293	0.294
otu	*OTU228*	0.00443	0.294
otu	*OTU299*	0.00443	0.294
otu	*OTU452*	0.00715	0.294
otu	*OTU3*	0.0105	0.294
otu	*OTU54*	0.0116	0.294
otu	*OTU139*	0.0117	0.294
otu	*OTU240*	0.0117	0.294
otu	*OTU286*	0.0117	0.294
otu	*OTU305*	0.0119	0.294
otu	*OTU356*	0.0119	0.294
otu	*OTU483*	0.0119	0.294
otu	*OTU17*	0.0125	0.294
otu	*OTU416*	0.0126	0.294
otu	*OTU472*	0.0126	0.294
otu	*OTU25*	0.0185	0.357
otu	*OTU174*	0.0187	0.357
otu	*OTU193*	0.0187	0.357
otu	*OTU296*	0.0187	0.357
otu	*OTU206*	0.0204	0.367

Showing top 40 taxa by BH-FDR q-value; full results in companion TSV file.

Supplementary Table 4: ANCOM and ANCOM-BC compositional analysis — all contrasts, significant taxa only (ANCOM-BC $q < 0.05$). 'ANCOM?' column gives the ANCOM W-statistic if also rejected by ANCOM.

**Contrast**	**Level**	**Taxon**	**$\log_2$FC**	**SE**	q	**ANCOM?**
B2_vs_B1	genus	*Ruminococcus*	-4.44	0.96	2.78e-04	W=15
B3_vs_B1	genus	*Bilophila*	+0.99	0.28	3.34e-02	–
B3_vs_B2	species	*Bacteroides_stercoris*	+4.48	0.83	7.96e-06	W=75
B3_vs_B2	genus	*Sutterella*	-3.54	0.71	6.28e-05	–
B3_vs_B2	phylum	*Fusobacteria*	+1.91	0.48	5.50e-04	–
B3_vs_B2	class	*Fusobacteriia*	+1.83	0.51	5.37e-03	–
B3_vs_B2	order	*Fusobacteriales*	+1.89	0.53	9.29e-03	–
CON_vs_RE	species	*Coprococcus_comes*	-2.05	0.48	2.10e-03	–
CON_vs_RE	species	*Bacteroides_plebeius*	-2.80	0.66	2.29e-03	–
CON_vs_RE	phylum	*Tenericutes*	-0.68	0.20	6.86e-03	–
CON_vs_RE	phylum	*Synergistetes*	-0.54	0.17	1.69e-02	–
CON_vs_RE	species	*Sutterella_wadsworthensis*	-2.08	0.56	1.78e-02	–
CON_vs_RE	class	*Mollicutes*	-0.73	0.24	3.32e-02	–
NHS3_2_vs_1	species	*Parasutterella_excrementihominis*	+0.57	0.15	1.22e-02	–
RE_vs_NRE	species	*Coprococcus_comes*	-2.38	0.48	6.98e-05	–
RE_vs_NRE	species	*Sutterella_wadsworthensis*	-2.80	0.65	1.64e-03	–
RE_vs_NRE	phylum	*Synergistetes*	-0.59	0.18	8.09e-03	–
RE_vs_NRE	genus	*Candidatus_Soleaferrea*	+0.38	0.11	4.01e-02	–
RE_vs_NRE	species	*Bacteroides_plebeius*	-2.86	0.81	4.63e-02	–

Supplementary Table 5: FDR-corrected Metastats: B2 (depression) vs. B1 (no comorbidity). No taxa survived FDR correction.

**Level**	**Taxon**	*P* **value**	**BH-FDR** *q*
class	*Betaproteobacteria*	0.0301	0.428
class	*Erysipelotrichia*	0.0475	0.428
family	*Pasteurellaceae*	0.0148	0.384
family	*Ruminococcaceae*	0.0163	0.384
family	*Alcaligenaceae*	0.0335	0.524
genus	*Ruminococcus*	0.00696	0.56
genus	*Haemophilus*	0.0145	0.56
genus	*Faecalibacterium*	0.016	0.56
order	*Pasteurellales*	0.0145	0.392
order	*Burkholderiales*	0.0293	0.396
order	*Erysipelotrichales*	0.0493	0.443
otu	*OTU126*	0.00637	0.871
otu	*OTU244*	0.00689	0.871
otu	*OTU118*	0.00878	0.871
otu	*OTU16*	0.00939	0.871
otu	*OTU107*	0.0142	1
otu	*OTU181*	0.03	1
otu	*OTU377*	0.03	1
otu	*OTU286*	0.0305	1
otu	*OTU25*	0.0414	1
otu	*OTU211*	0.0448	1
otu	*OTU218*	0.0448	1
otu	*OTU340*	0.0448	1
otu	*OTU369*	0.0474	1
phylum	*Proteobacteria*	0.0346	0.346
species	*Haemophilus_parainfluenzae*	0.0115	0.767
species	*Blautia_sp.*	0.0171	0.767
species	*Megasphaera_micronuciformis*	0.03	0.767
species	*Ruminococcus_sp._N15.MGS-57*	0.0477	0.767

Supplementary Table 6: FDR-corrected Metastats: B3 (anxiety+depression) vs. B1 (no comorbidity). No taxa survived FDR correction.

**Level**	**Taxon**	P **value**	**BH-FDR** q
class	*Epsilonproteobacteria*	0.0419	0.637
family	*Campylobacteraceae*	0.0419	1
genus	*Bilophila*	0.00805	0.492
genus	*Cloacibacillus*	0.00936	0.492
genus	*Erysipelatoclostridium*	0.0288	0.55
genus	*Intestinibacter*	0.0377	0.55
genus	*Parasutterella*	0.0408	0.55
genus	*Peptostreptococcus*	0.0419	0.55
genus	*Campylobacter*	0.0419	0.55
genus	*Johnsonella*	0.0419	0.55
order	*Campylobacterales*	0.0419	0.896
otu	*OTU332*	0.000454	0.0842
otu	*OTU340*	0.000454	0.0842
otu	*OTU250*	0.00164	0.203
otu	*OTU165*	0.00499	0.313
otu	*OTU265*	0.00561	0.313
otu	*OTU273*	0.00561	0.313
otu	*OTU347*	0.00591	0.313
otu	*OTU172*	0.00895	0.386
otu	*OTU268*	0.00936	0.386
otu	*OTU5*	0.011	0.408
otu	*OTU295*	0.0141	0.476
otu	*OTU274*	0.0165	0.51
otu	*OTU275*	0.0204	0.527
otu	*OTU302*	0.0213	0.527
otu	*OTU359*	0.0213	0.527
otu	*OTU285*	0.0258	0.597
otu	*OTU174*	0.0278	0.597
otu	*OTU49*	0.029	0.597
otu	*OTU30*	0.034	0.633
otu	*OTU24*	0.0342	0.633
otu	*OTU287*	0.0419	0.633
otu	*OTU351*	0.0419	0.633
otu	*OTU365*	0.0419	0.633
otu	*OTU336*	0.042	0.633
otu	*OTU102*	0.045	0.633
otu	*OTU240*	0.0456	0.633
otu	*OTU144*	0.0492	0.633
otu	*OTU244*	0.0498	0.633
species	*Peptoniphilus_lacrimalis*	0.00561	0.255

Showing top 40 taxa by BH-FDR q-value; full results in companion TSV file.

Supplementary Table 7: FDR-corrected Metastats: B3 (anxiety+depression) vs. B2 (depression only). No taxa survived FDR correction.

**Level**	**Taxon**	P **value**	**BH-FDR** q
family	*Acidaminococcaceae*	0.0118	0.554
genus	*Phascolarctobacterium*	0.0132	0.668
genus	*Sutterella*	0.0216	0.668
genus	*Ruminococcus*	0.0262	0.668
genus	*Desulfovibrio*	0.0318	0.668
genus	*Cloacibacillus*	0.0318	0.668
genus	*Adlercreutzia*	0.0392	0.685
genus	*Escherichia*	0.0499	0.749
otu	*OTU5*	0.00362	0.922
otu	*OTU172*	0.00805	0.922
otu	*OTU20*	0.0144	0.922
otu	*OTU97*	0.0156	0.922
otu	*OTU238*	0.0237	0.922
otu	*OTU25*	0.03	0.922
otu	*OTU244*	0.0318	0.922
otu	*OTU268*	0.0318	0.922
otu	*OTU14*	0.0319	0.922
otu	*OTU182*	0.0322	0.922
otu	*OTU273*	0.0332	0.922
otu	*OTU76*	0.0394	0.922
otu	*OTU350*	0.0401	0.922
otu	*OTU205*	0.0407	0.922
otu	*OTU142*	0.0444	0.922
otu	*OTU302*	0.0461	0.922
species	*Bacteroides_stercoris*	0.00355	0.44
species	*gut_metagenome*	0.0158	0.705
species	*Cloacibacillus_porcorum*	0.0318	0.705
species	*Ruminococcus_sp._N15.MGS-57*	0.0319	0.705
species	*[Bacteroides]_coagulans*	0.0332	0.705
species	*Escherichia_coli*	0.0341	0.705
species	*uncultured_Lachnospiraceae_bacterium*	0.0425	0.753

Supplementary Table 8: FDR-corrected Metastats: NHS3 seizure severity pairwise comparisons (all). Bold q indicates FDR-significant.

**Level**	**Taxon**	P **value**	**BH-FDR** q
class	*unclassified*	0.000999	**0.018**
class	*Lentisphaeria*	0.0106	0.192
class	*Mollicutes*	0.0465	0.418
family	*Eubacteriaceae*	0.00336	0.115
family	*Comamonadaceae*	0.00491	0.115
family	*Victivallaceae*	0.0106	0.5
family	*Eubacteriaceae*	0.011	0.519
family	*Leptotrichiaceae*	0.0353	0.553
genus	*Desulfovibrio*	0.000999	0.0529
genus	*Lactococcus*	0.000999	0.0529
genus	*Oscillibacter*	0.000999	0.106
genus	*Eubacterium*	0.00336	0.26
genus	*Comamonas*	0.00491	0.26
genus	*Anaerotruncus*	0.00799	0.282
genus	*Eubacterium*	0.011	0.353
genus	*Cloacibacillus*	0.0167	0.353
genus	*Johnsonella*	0.0167	0.353
genus	*Victivallis*	0.0106	0.461
genus	*Lactococcus*	0.0131	0.461
genus	*Peptostreptococcus*	0.0204	0.54
genus	*Pyramidobacter*	0.0327	0.54
genus	*Peptostreptococcus*	0.0417	0.54
genus	*Caproiciproducens*	0.0417	0.54
genus	*Shuttleworthia*	0.0496	0.54
genus	*Alloprevotella*	0.0294	0.623
genus	*Cloacibacillus*	0.0268	0.654
genus	*Sneathia*	0.0353	0.654
genus	*Senegalimassilia*	0.04	0.654
genus	*unclassified*	0.045	0.654
genus	*Olsenella*	0.0465	0.754
order	*Victivallales*	0.0106	0.287
otu	*OTU39*	0.000999	**0.0232**
otu	*OTU95*	0.000999	**0.0232**
otu	*OTU152*	0.000999	**0.0232**
otu	*OTU178*	0.000999	**0.0232**
otu	*OTU180*	0.000999	**0.0232**
otu	*OTU199*	0.000999	**0.0232**
otu	*OTU220*	0.000999	**0.0232**
otu	*OTU233*	0.000999	**0.0232**
otu	*OTU243*	0.000999	**0.0232**

Showing top 40 taxa by BH-FDR q-value; full results in companion TSV file.

2 Supplementary Figures

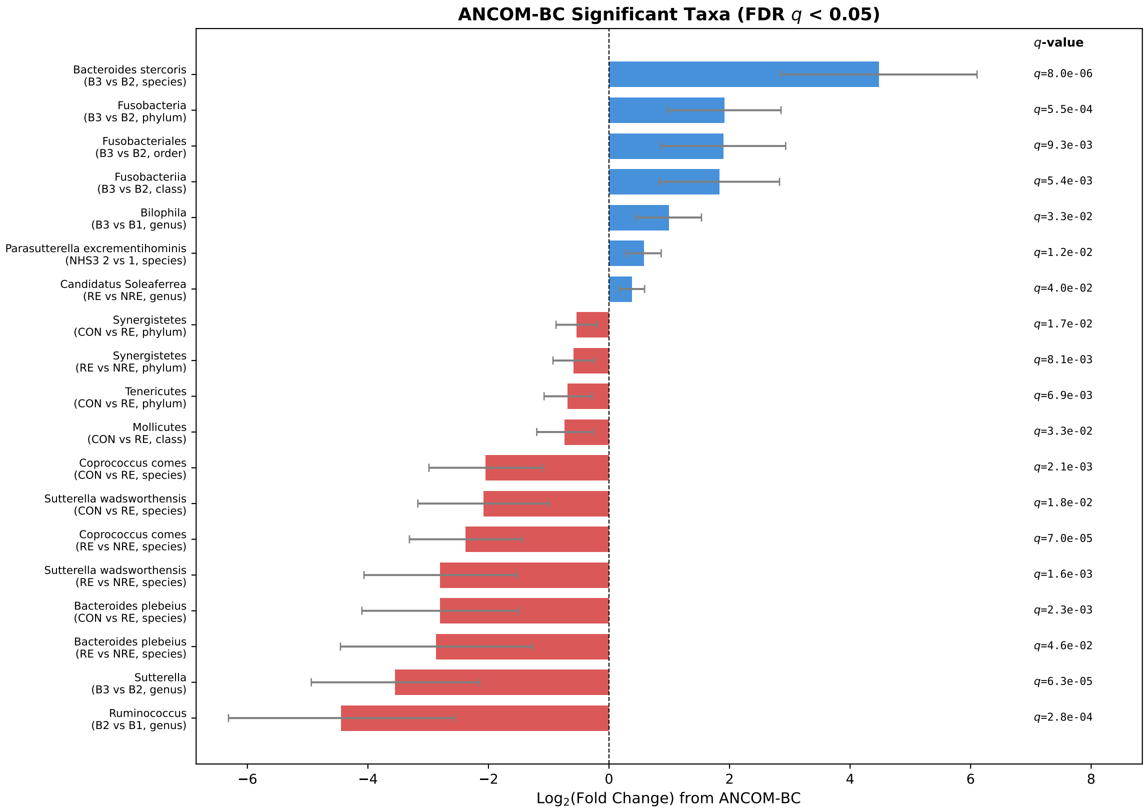

Supplementary Figure 1: **Supplementary Figure S1.** Forest plot of ANCOM-BC-significant taxa (FDR $q < 0.05$) across all contrasts. Red bars: depletion in first-named group; blue bars: enrichment. Error bars show approximate 95% CI from standard error. Right column gives FDR-adjusted q-values.

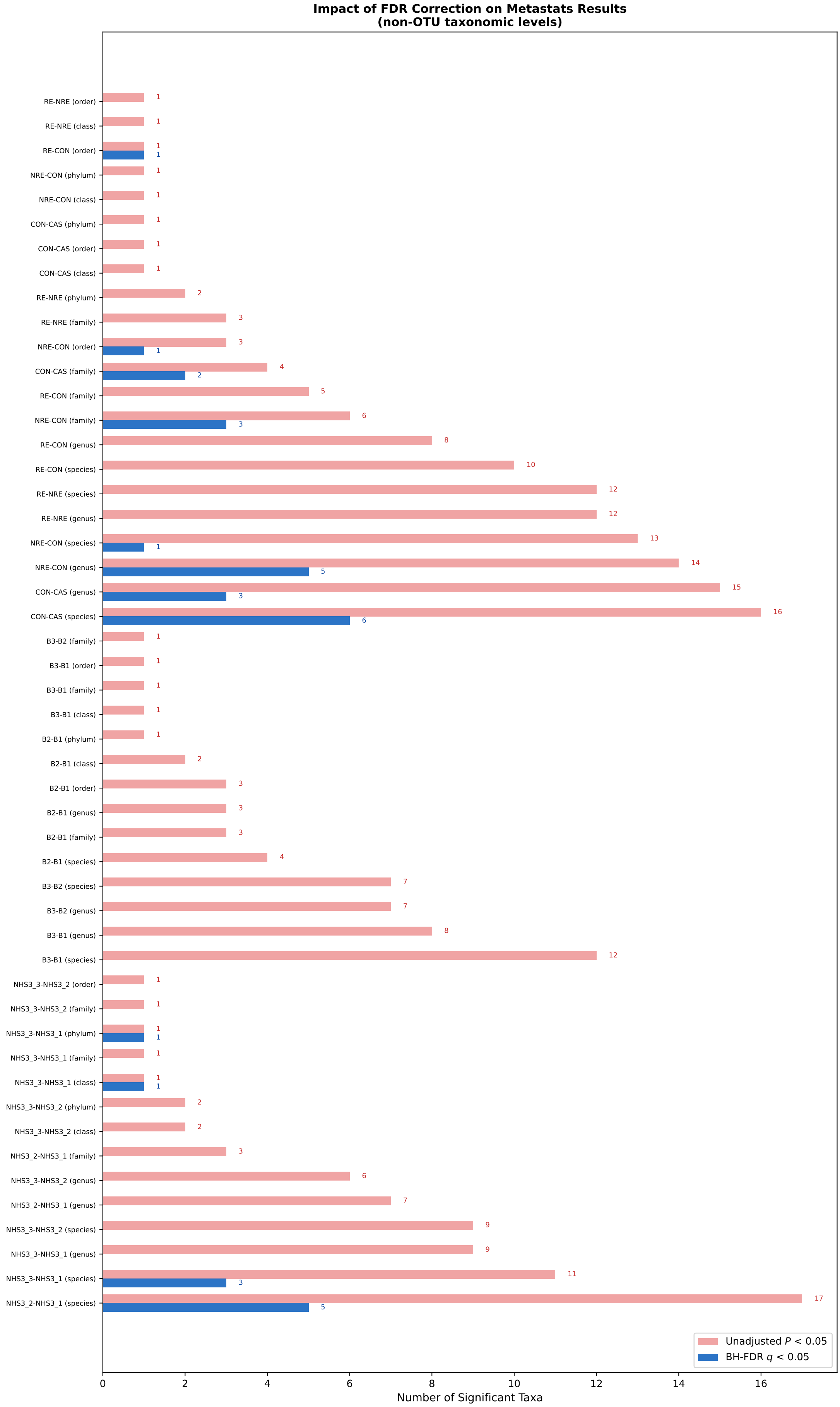

Supplementary Figure 2: **Supplementary Figure S2.** Impact of BH-FDR correction on Metastats results across all cohorts and taxonomic ranks (excluding OTU-level). Pink bars show the number of taxa significant at unadjusted $P < 0.05$; blue bars show those surviving FDR at $q < 0.05$.

Supplementary Figure S3

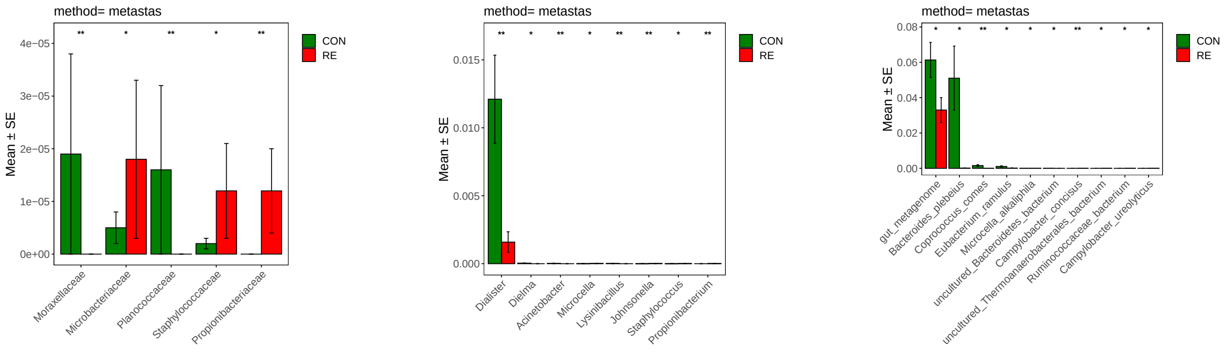

(a) (b) (c)

Supplementary Figure 3: Figure S3. CON vs RE — Metastats differential analysis at family, genus, species levels.

Supplementary Figure S4

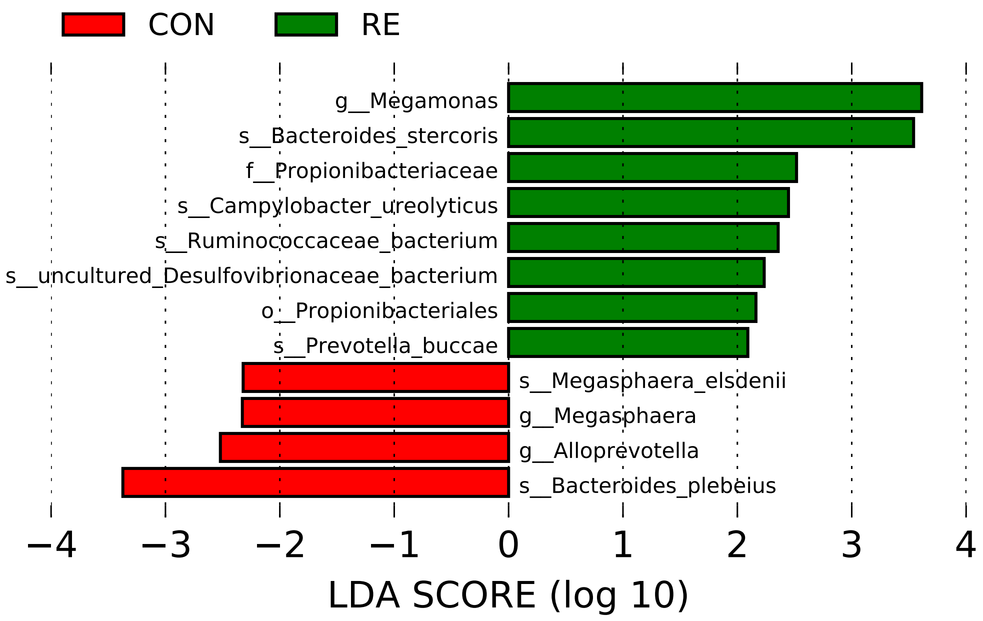

Supplementary Figure 4: Figure S4. CON vs RE — LEfSe LDA score plot.

Supplementary Figure S5

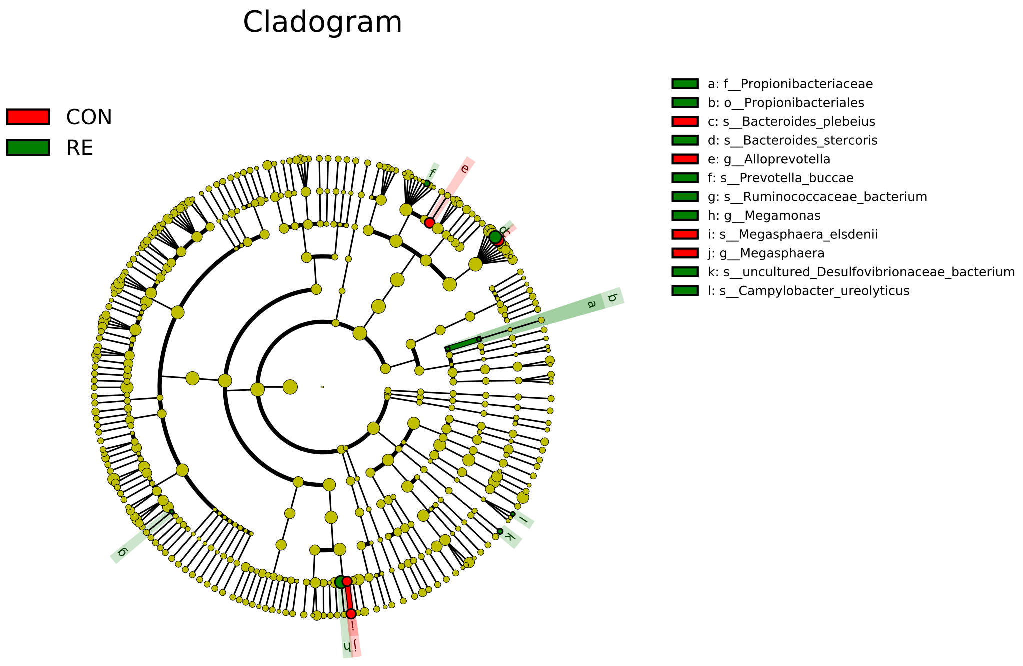

Supplementary Figure 5: Figure S5. CON vs RE — LEfSe cladogram.

Supplementary Figure S6

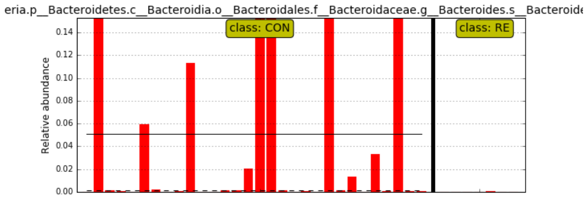

(a)

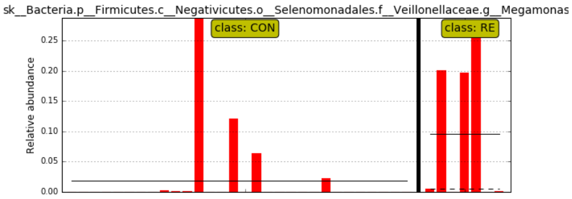

(b)

Supplementary Figure 6: Figure S6. CON vs RE — LEfSe bar plots.

Supplementary Figure S7

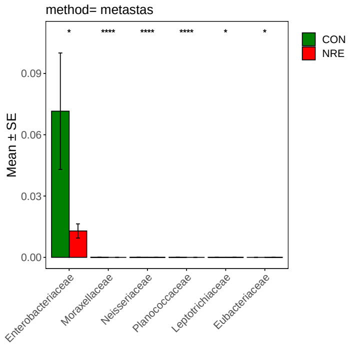

(a)

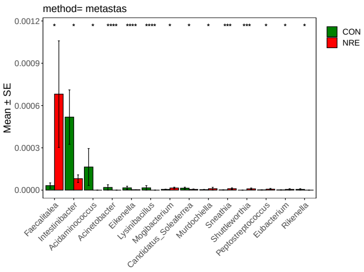

(b)

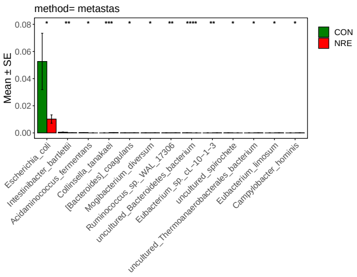

(c)

Supplementary Figure 7: Figure S7. CON vs NRE — Metastats differential analysis.

Supplementary Figure S8

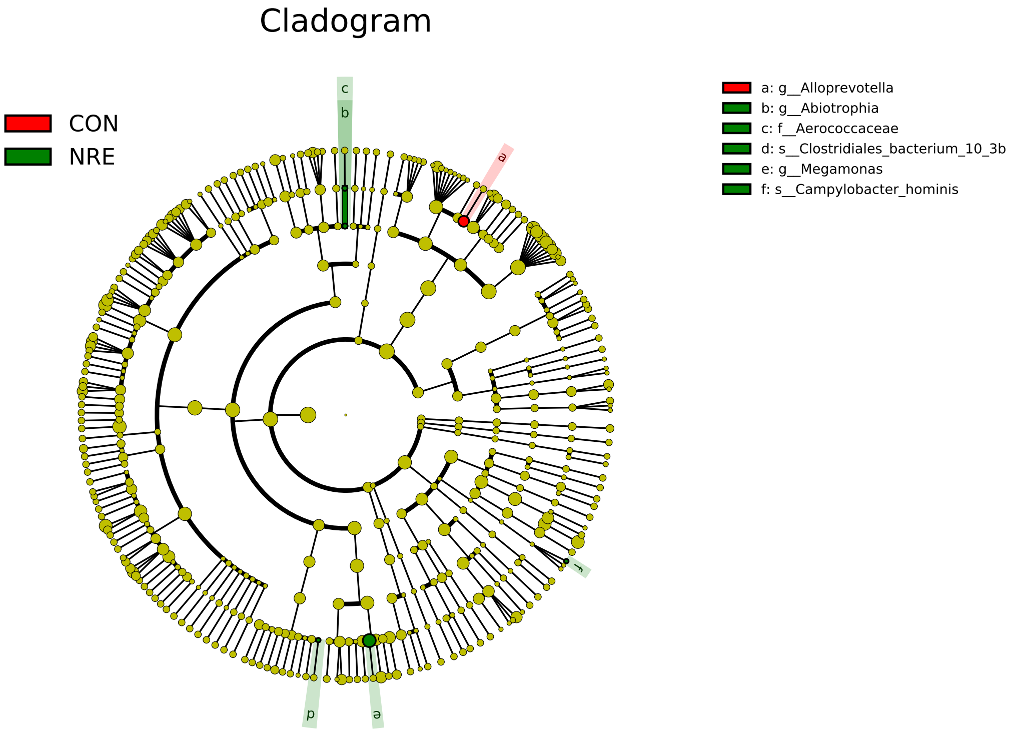

Supplementary Figure 8: Figure S8. CON vs NRE — LEfSe cladogram.

Supplementary Figure S9

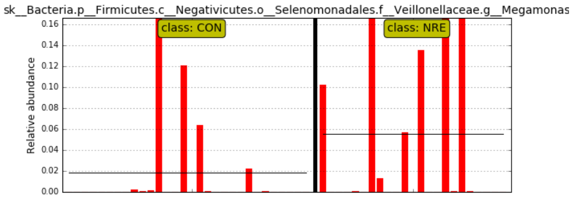

(a)

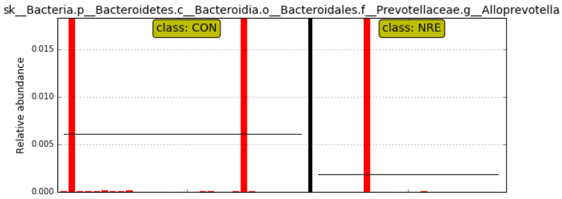

(b)

Supplementary Figure 9: Figure S9. CON vs NRE — LEfSe bar plots.

Supplementary Figure S10

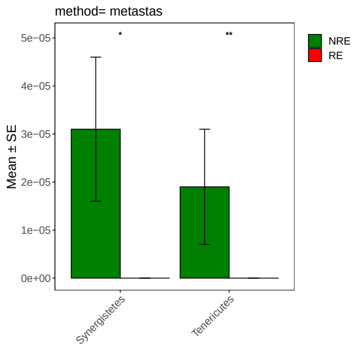

(a)

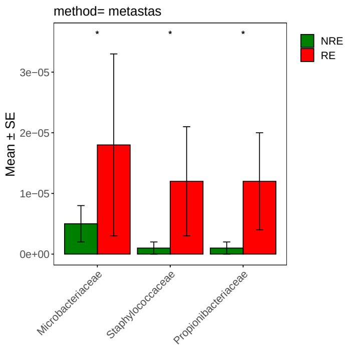

(b)

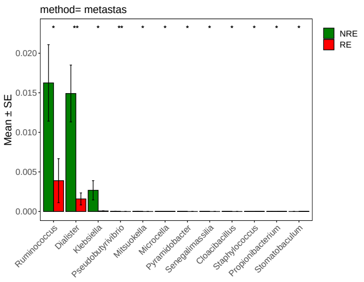

(c)

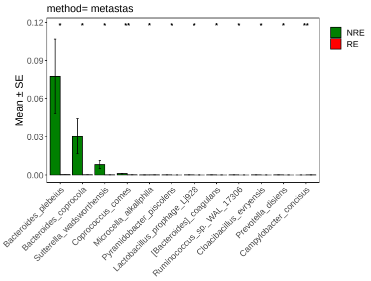

(d)

Supplementary Figure 10: Figure S10. RE vs NRE — Metastats differential analysis.

Supplementary Figure S11

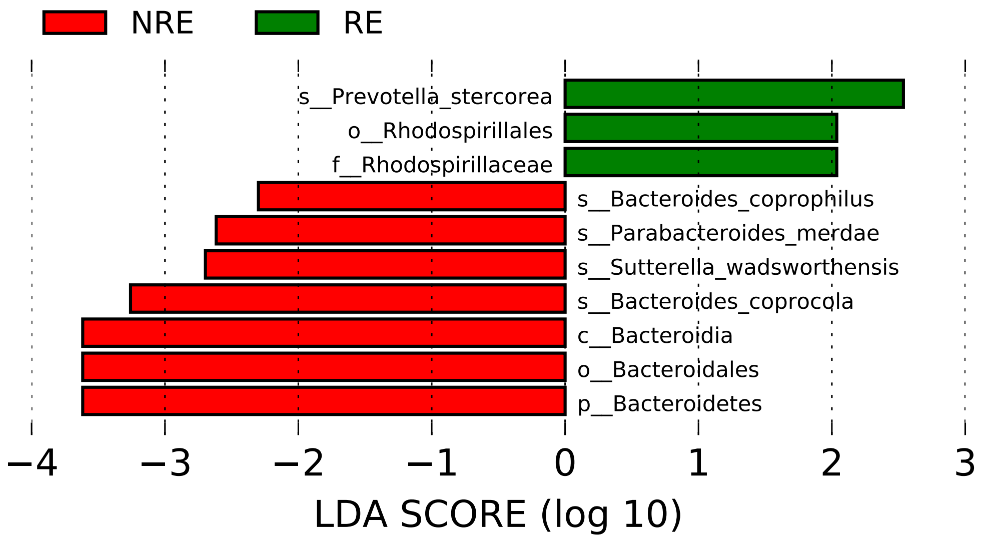

Supplementary Figure 11: Figure S11. RE vs NRE — LEfSe LDA score plot.

Supplementary Figure S12

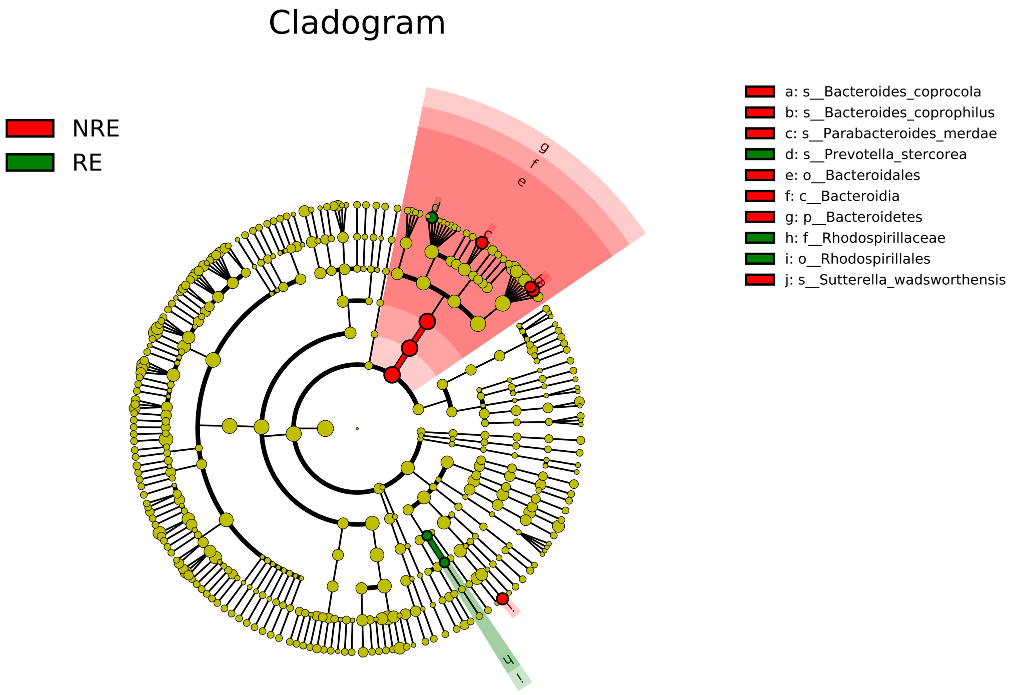

Supplementary Figure 12: Figure S12. RE vs NRE — LEfSe cladogram.

Supplementary Figure S13

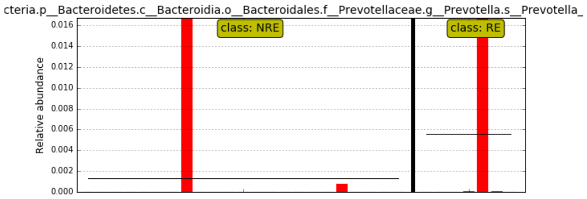

(a)

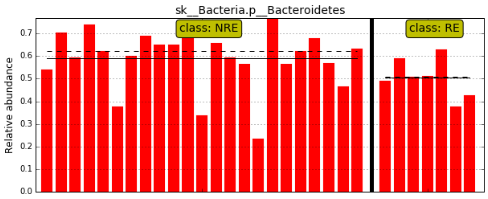

(b)

Supplementary Figure 13: Figure S13. RE vs NRE — LEfSe bar plots.

Supplementary Figure S14

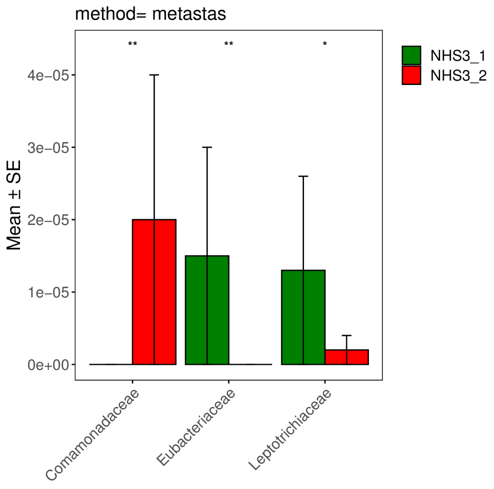

(a)

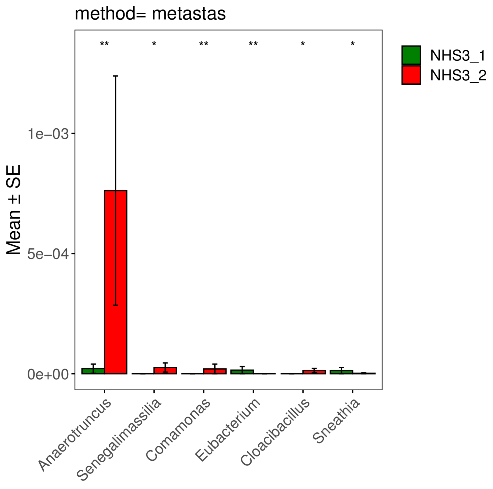

(b)

Supplementary Figure 14: Figure S14. NHS3 severity — Metastats differential analysis (part 1).

Supplementary Figure S15

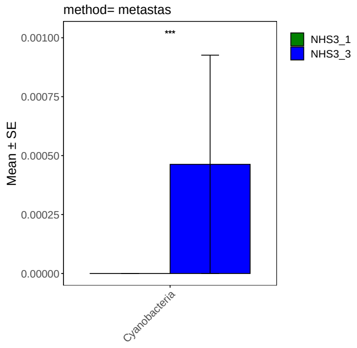

(a)

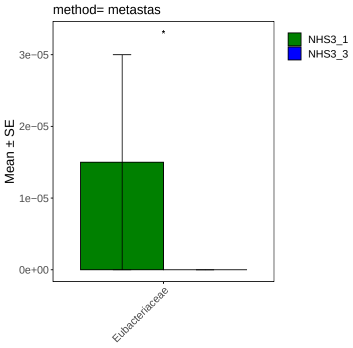

(b)

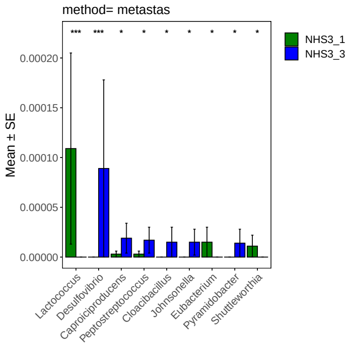

(c)

Supplementary Figure 15: Figure S15. NHS3 severity — Metastats differential analysis (part 2).

Supplementary Figure S16

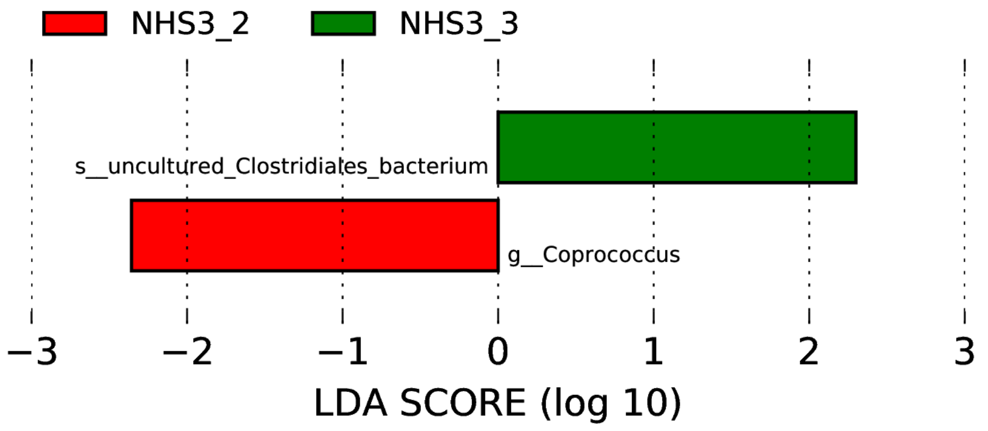

Supplementary Figure 16: Figure S16. NHS3 severity — LEfSe LDA score plot.

Supplementary Figure S17

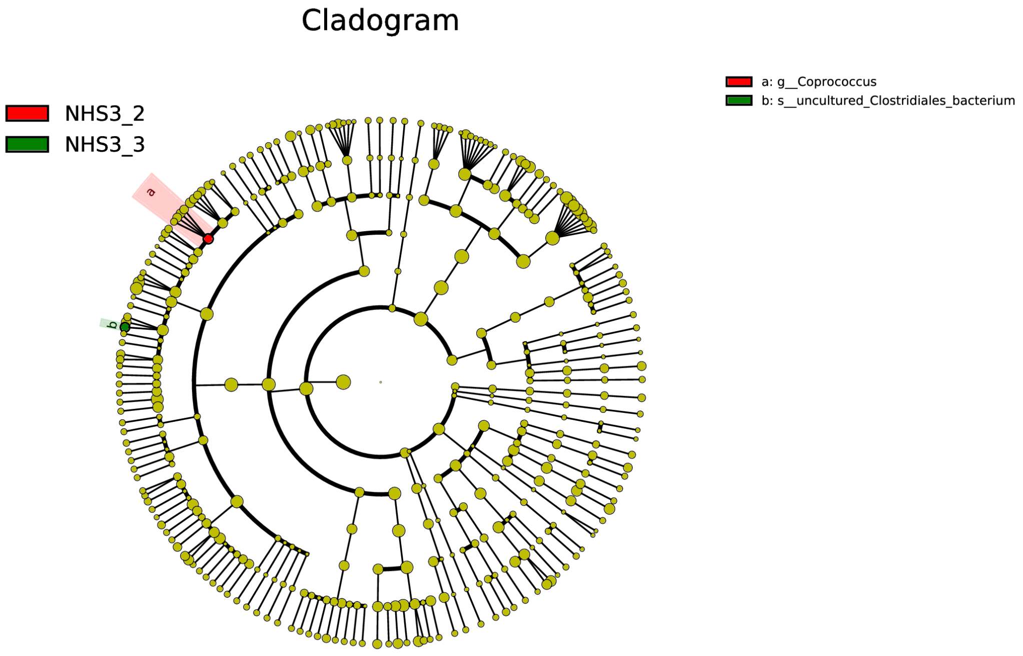

Supplementary Figure 17: Figure S17. NHS3 severity — LEfSe cladogram.

Supplementary Figure S18

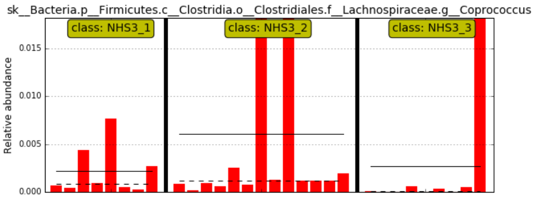

(a)

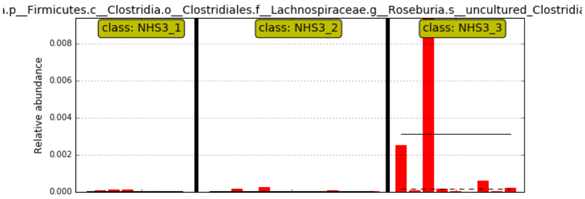

(b)

Supplementary Figure 18: Figure S18. NHS3 severity — LEfSe bar plots.

Supplementary Figure S19

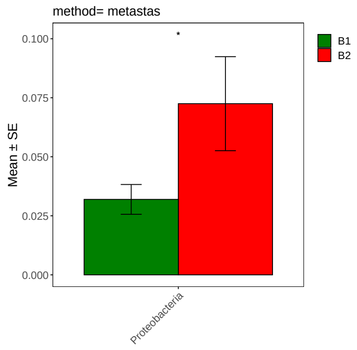

(a)

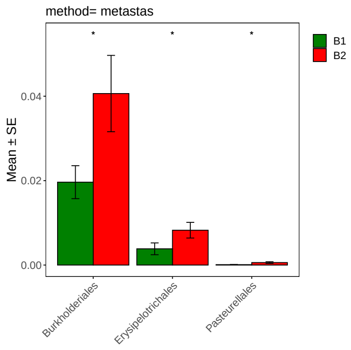

(b)

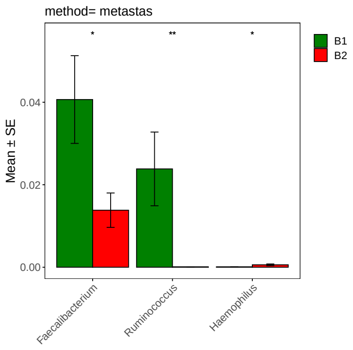

(c)

Supplementary Figure 19: Figure S19. B2 vs B1 — Metastats differential analysis.

Supplementary Figure S20

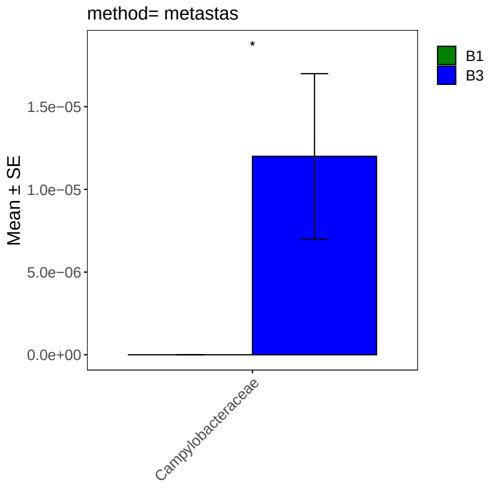

(a)

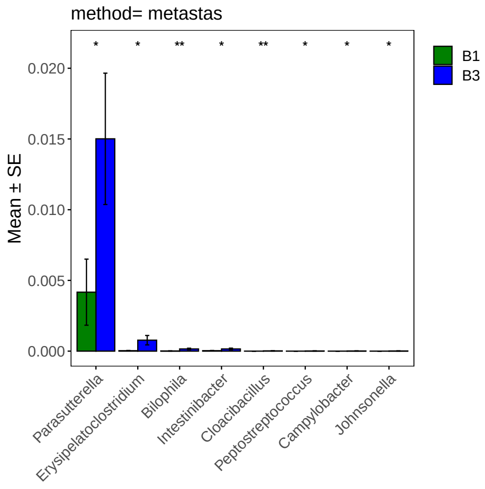

(b)

Supplementary Figure 20: Figure S20. B3 vs B1 — Metastats differential analysis.

Supplementary Figure S21

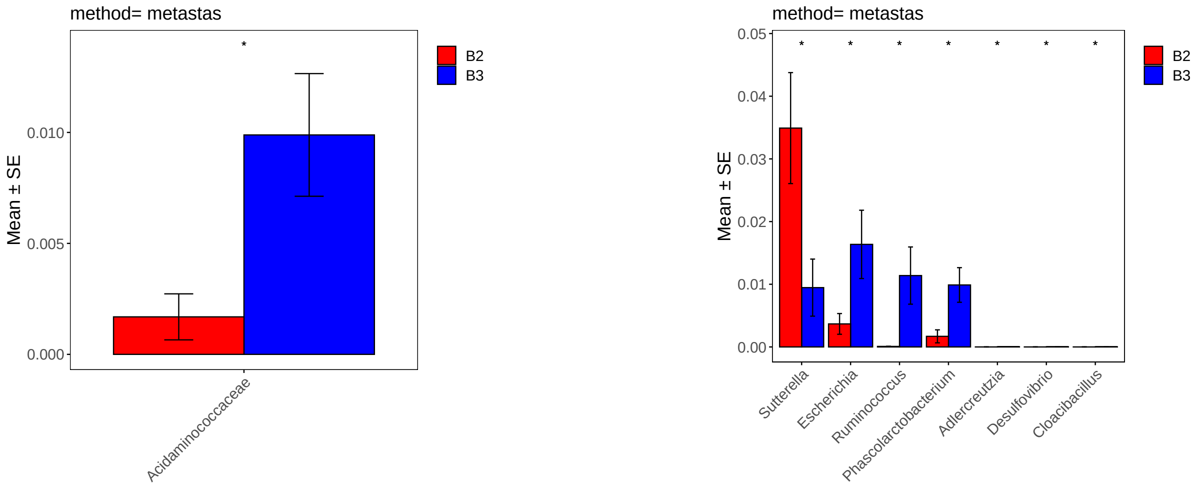

(a) (b)

Supplementary Figure 21: Figure S21. B3 vs B2 — Metastats differential analysis.

Supplementary Figure S22

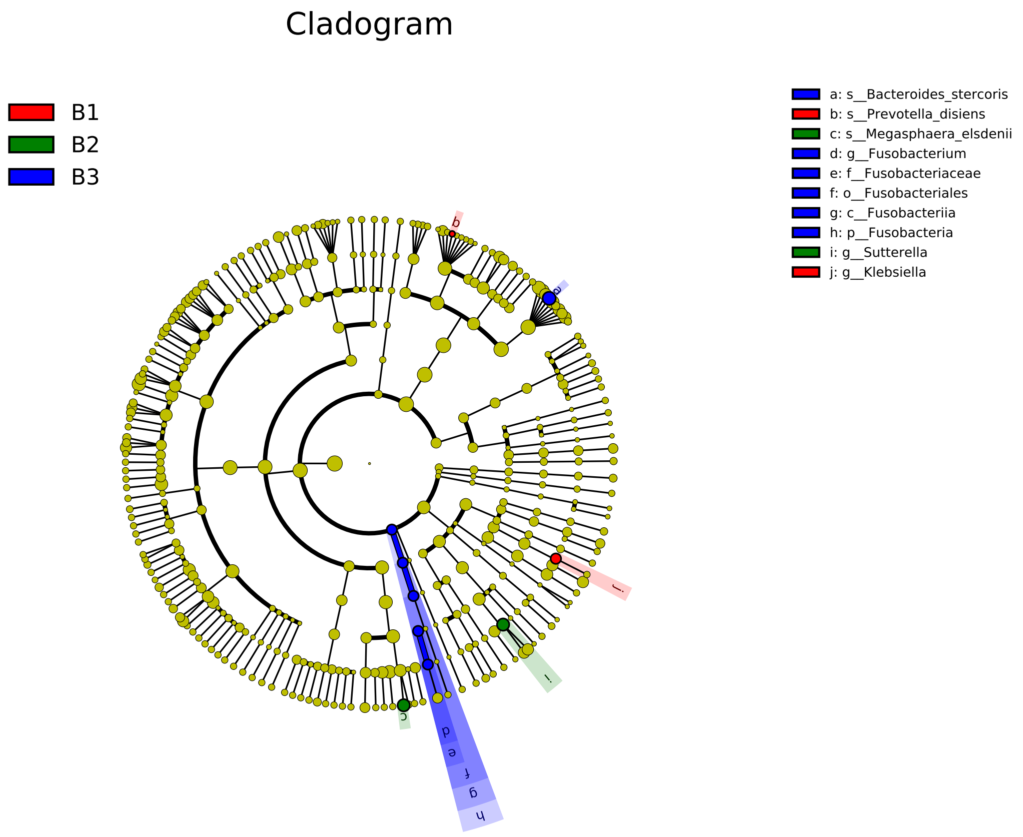

Supplementary Figure 22: Figure S22. B groups — LEfSe cladogram.

Supplementary Figure S23

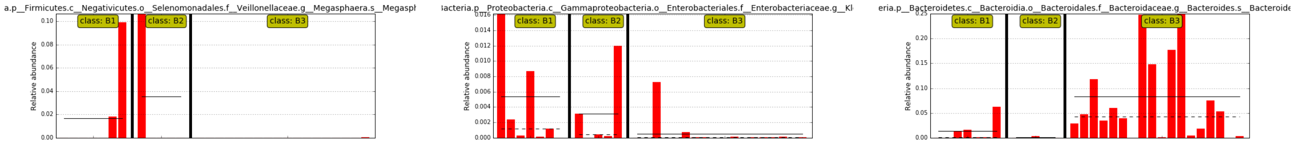

(a) (b) (c)

Supplementary Figure 23: Figure S23. B groups — LEfSe bar plots.
